# Modeling holo-ACP:DH and holo-ACP:KR complexes of modular polyketide synthases: a docking and molecular dynamics study

**DOI:** 10.1186/1472-6807-12-10

**Published:** 2012-05-28

**Authors:** Swadha Anand, Debasisa Mohanty

**Affiliations:** 1 Bioinformatics Center, National Institute of Immunology, Aruna Asaf Ali Marg, New Delhi, 110067, India

**Keywords:** Molecular dynamics, Protein-ligand docking, Protein-protein interaction, Substrate binding site, Evolutionary conservation, Modular polyketide synthase, Dehydratase domain, Ketoreductase domain

## Abstract

**Background:**

Modular polyketide synthases are multifunctional megasynthases which biosynthesize a variety of secondary metabolites using various combinations of dehydratase (DH), ketoreductase (KR) and enoyl-reductase (ER) domains. During the catalysis of various reductive steps these domains act on a substrate moiety which is covalently attached to the phosphopantetheine (P-pant) group of the holo-Acyl Carrier Protein (holo-ACP) domain, thus necessitating the formation of holo-ACP:DH and holo-ACP:KR complexes. Even though three dimensional structures are available for DH, KR and ACP domains, no structures are available for DH or KR domains in complex with ACP or substrate moieties. Since Ser of holo-ACP is covalently attached to a large phosphopantetheine group, obtaining complexes involving holo-ACP by standard protein-protein docking has been a difficult task.

**Results:**

We have modeled the holo-ACP:DH and holo-ACP:KR complexes for identifying specific residues on DH and KR domains which are involved in interaction with ACP, phosphopantetheine and substrate moiety. A novel combination of protein-protein and protein-ligand docking has been used to first model complexes involving apo-ACP and then dock the phosphopantetheine and substrate moieties using covalent connectivity between ACP, phosphopantetheine and substrate moiety as constraints. The holo-ACP:DH and holo-ACP:KR complexes obtained from docking have been further refined by restraint free explicit solvent MD simulations to incorporate effects of ligand and receptor flexibilities. The results from 50 ns MD simulations reveal that substrate enters into a deep tunnel in DH domain while in case of KR domain the substrate binds a shallow surface exposed cavity. Interestingly, in case of DH domain the predicted binding site overlapped with the binding site in the inhibitor bound crystal structure of FabZ, the DH domain from *E.Coli* FAS*.* In case of KR domain, the substrate binding site identified by our simulations was in proximity of the known stereo-specificity determining residues.

**Conclusions:**

We have modeled the holo-ACP:DH and holo-ACP:KR complexes and identified the specific residues on DH and KR domains which are involved in interaction with ACP, phosphopantetheine and substrate moiety. Analysis of the conservation profile of binding pocket residues in homologous sequences of DH and KR domains indicated that, these results can also be extrapolated to reductive domains of other modular PKS clusters.

## Backgrounds

Polyketides are secondary metabolites that constitute a major class of pharmaceutically important compounds. The biosynthesis is catalyzed by multi-functional enzymes using an assembly line mechanism. The various domains in these multi-functional enzymes are arranged as modules where each module catalyzes the addition of one extender unit. Each module is constituted by a set of domains responsible for adding and modifying the extender unit. The biosynthetic intermediate is covalently attached to Acyl Carrier Protein (ACP) domain by a thioester linkage and is transferred from one catalytic site to another. The condensation of extender unit with the elongating polyketide chain is brought about by ketosynthase (KS) and Acyltransferase (AT) domain. The beta-keto product formed by action of KS and AT domain can be further modified by a combination of ketoreductase (KR), dehydratase (DH) and enoyl-reductase (ER) domains to form a hydroxyl group (KR only), a double bond (KR and DH) or a single bond (DH, ER and KR) containing moiety [1]. Although, earlier studies [2-6] have attempted to relate the sequences of DH and KR domains to their function, till recently, no crystal structures were available for these reductive domains from modular PKSs. The only structural information was available for Type II FAS DH [7-9] and Type II PKS KR [10-15] domains and it was presumed that the Type I PKS domains also utilized similar catalytic mechanisms as the type II enzymes. The recent availability of the crystal structures of these domains from Type I PKS [5,16-20] has opened up an opportunity to utilize structural information to understand substrate recognition by these reductive domains and the mechanism of reactions catalyzed by them.

**Figure 1:**
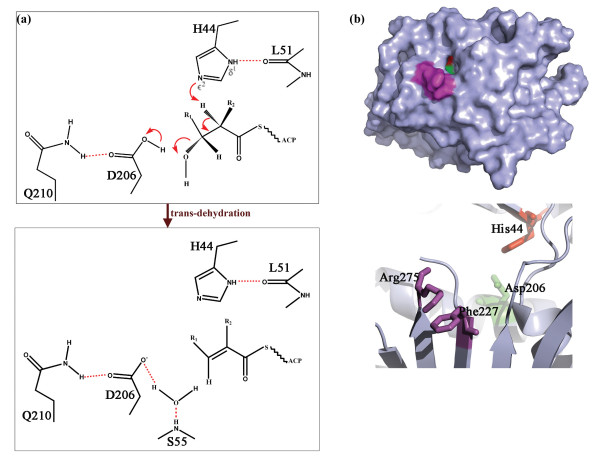
**a) Schematic diagram depicting the mechanism of reaction catalyzed by DH domain.b**) The Arg 275 and Phe 227 shown in magenta are hypothesized to be involved in ACP binding while His 44 (red) and Asp 206 (green) are catalytic residues.

Recently the structure of dehydratase from the fourth module of erythromycin synthase was solved by Keatinge Clay [19]. The structure showed that the DH domain possesses a double hot dog fold and the conserved hydrophobic residues on the DH surface were proposed to be responsible for its interaction with other domains like ACP. Molecular modeling studies have also predicted how the alpha-hydrogen and beta-hydroxyl group of a polyketide substrate might be able to interact with the catalytic histidine and aspartic acid in the DH active site [19]. The structure shows that the catalytic His is provided by one half of double hot dog fold while the catalytic Asp is provided by the other. The structure also reveals the orientation of various catalytic residues and hence, provides an insight into mechanistic details. It shows that the catalytic His 44 is oriented to interact with substrate through van der Waals contacts with Pro53 and Hydrogen-bonding with Leu51. The role of general acid is usually played by Glu in type II PKSs but it is replaced by Asp in type I PKSs. The catalytic Asp is shown to make H-bonding interaction with Gln 210 which aids in its positioning in the active site. The DH is hypothesized to catalyze dehydration by abstraction of proton by catalytic His 44 and donation of proton by catalytic Asp 206 to the β-hydroxyl group thereby yielding a water molecule. The electrons from Cα-H bond migrate to form Cα-Cβ trans double bond after abstraction of Cα proton by His 44 (Figure 1a). The natural substrate for DH domain of fourth module of erythromycin synthase is (2R,3R,4R,6R,7S,8S,9R)-3,7,9-trihydroxy-5-oxo-2,4,6,8-tetramethyl undecanoate holo EryACP4 thioester [19]. It was shown by mutagenesis studies that a nearly invariant Arg 275, when mutated to Asp led to a lesser production of product 6-dEB, indicating its role in ACP recognition. [19] This residue being close to active site tunnel further indicated that it may form a salt bridge with Asp in DSL motif of cognate ACP. Another highly conserved residue F227 is also implicated to be involved in interaction with ACP domain. These residues are present close to active site entrance (Figure 1b), which is a narrow tunnel leading to catalytic residues His 44 (red) and Asp 206 (green) (Figure 1b). The structures of dehydratases from the curacin PKS gene cluster have also been solved by Akey *et al*[18]. These structures also confirm to double hot dog fold and the arrangement of active site residues is similar to erythromycin DH.

Ketoreductases reduce the beta carboxyl to a hydroxyl group and thereby introduce a chiral center in the polyketide products. Thus, they are responsible for determining the chirality of the polyketide product [6]. Earlier studies have identified sequence motifs predictive of hydroxyl group stereochemistry brought about by KR domains. KRs that produce a hydroxyl group of “S” stereochemistry are called as A-type KRs and have a conserved tryptophan residue while B-type KRs containing a LDD motif produce a hydroxyl group of “R” stereochemistry [6]. The KRs have been shown to maintain stereo-control in isolation also and thus, their role in determining alpha substituent chirality was proposed [5]. This brings out the possibility of six types of KRs with characteristic motifs. As shown by Keatinge-Clay, A1 and B1 KRs catalyze reduction but no epimerization while A2 and B2 KRs catalyze epimerization as well as reduction [5].

**Figure 2:**
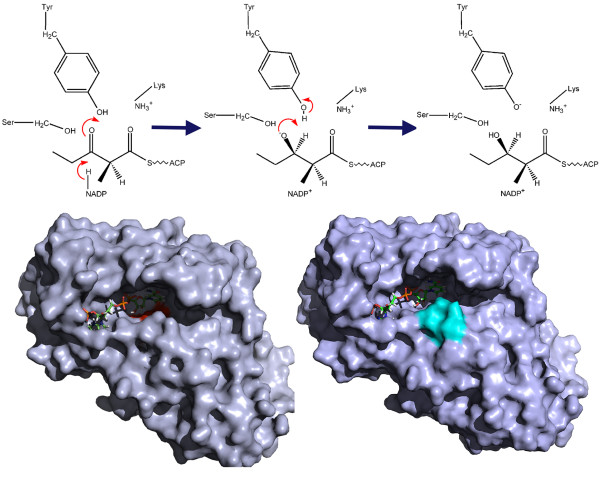
**Schematic diagram depicting the mechanism of reaction catalyzed by KR domain.** The catalytic residues Tyr 1813 and Ser 1800 are marked in red in left panel along with the NADPH shown as stick representation. The LDD motif has been marked in cyan color in right panel.

Recently, Keatinge-Clay & Stroud have solved the crystal structures of KR domain from first module of erythromycin synthase which constituted two sub-domains, the structural and catalytic sub-domain, both showing Rossmann fold similar to SDR family of enzymes, in spite of low sequence similarity [20]. The structural sub-domain is formed by the region earlier referred to as the linker preceding the KR domains. However, only the catalytic sub-domain possesses the conserved catalytic residues and the boundary for catalytic KR sub-domain was also shown to extend by ~70 amino acid residues into the linker region earlier called as KR-ACP linker. The mechanism of catalysis by KR has also been proposed where a substrate binds to the cofactor NADPH bound KR domain. The β-carbonyl interacts with S1800 and Y1813 and NADPH hydride attacks the carbonyl group from below. The oxygen hereby, accepts a proton from Y1813, resulting in a R stereochemistry for the β-hydroxyl group (Figure 2a). The mechanism of epimerization of substrate by the KR has also been proposed. The catalytic Y1813 acts as a base and picks acidic hydrogen of the diketide to form an enolate intermediate. This proton is taken back by enolate oxygen after which the enolized isomer of the diketide is released from the KR. A mixture of the original diketide and the epimerized diketide will be obtained if an uncatalyzed tautomerization back to the keto form occurs and KR can again accept the original diketide until it gets epimerized. The binding site has two entrance points, one where the NADPH is bound and the other entrance is formed by LDD motif which is shown to be conserved in B-type KR domains. This LDD motif has been proposed to guide the substrate entry through the second entrance (Figure 2b**)**. The structure for the Tylosin KR has also been reported by Keatinge Clay recently [5]. Tylosin KR, unlike the erythromycin KR catalyzes only reduction of the beta-hydroxyl but not epimerization of the alpha-substituent group. The structure showed similar fold to that of EryKR but a detailed structural and sequence analysis revealed sequence features which could help in distinguishing these two types of KRs. The crystal structure of A-type KR from second module of amphotericin PKS cluster has revealed the role of conserved tryptophan in determining S-stereochemistry for beta-hydroxyl group [17]. Recently, the structures of stand-alone KR domains from Type II PKS Hedamycin and Actinorhodin were solved. Biochemical studies guided by analysis of these structures have revealed that in addition to the “XGG” motif, other residues in the binding site pocket of the Type II ketoreductases can be responsible for determining regio- and stereo-specificity [14,15].

The DH and KR domains carry out their catalytic activity on the ACP bound substrate moiety. Even though, the crystal structures are available for DH and KR domains of modular PKS [21], no structural details are available for binding of the ACP domain to these reductive domains. The details about the interactions of ACP bound P-pant and substrate moieties with these domains are also not elucidated in details, as of now. In this study, we have made an attempt to model the holo-ACP:DH and holo-ACP:KR complexes and analyze P-pant as well as substrate binding sites on DH and KR domains to understand the mechanistic details of catalysis by these domains. In order to model holo-ACP:DH and holo-ACP:KR complexes, first apo-ACP:DH and apo-ACP:KR complexes have been modeled by protein-protein docking. This is followed by docking of P-pant and substrate moiety onto the apo-ACP:DH and apo-ACP:KR complexes. The necessary covalent bonds have been made between Ser of ACP, P-pant and substrate moiety to generate holo-ACP:DH and holo-ACP:KR complexes. In both the protein-protein as well as protein ligand docking, the solutions obtained have been filtered based on functional constraints arising from reactions catalyzed by these domains. Various recent studies have suggested that protein ligand docking followed by MD simulation studies to provide protein flexibility, can give useful information about critical residues involved in protein-ligand interactions [22-25]. The complexes obtained using docking studies by above-mentioned protocol have been further refined using MD simulations to incorporate effects of ligand and receptor flexibilities. Putative binding pocket residues of DH and KR domains involved in interactions with ACP, phosphopantetheine and substrate moiety have been identified from modeled apo-ACP:DH and apo-ACP:KR complexes and their conservation profile has also been analyzed.

## Results and discussion

### Modeling and analysis of substrate bound holo-ACP:DH complex

**Figure 3:**
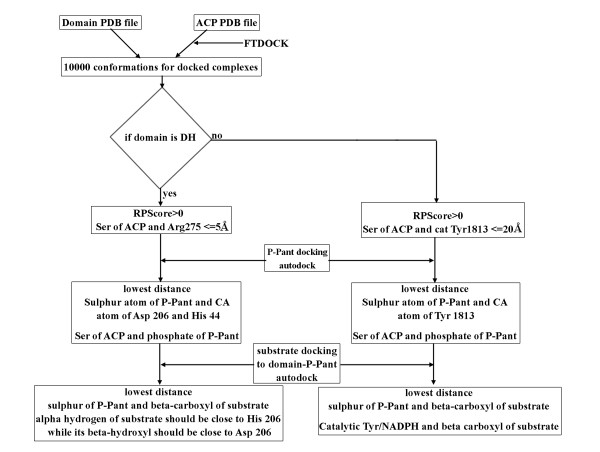
**Protocol for*in silico*modeling of substrate bound holo ACP-DH and holo ACP-KR complexes.**

**Figure 4:**
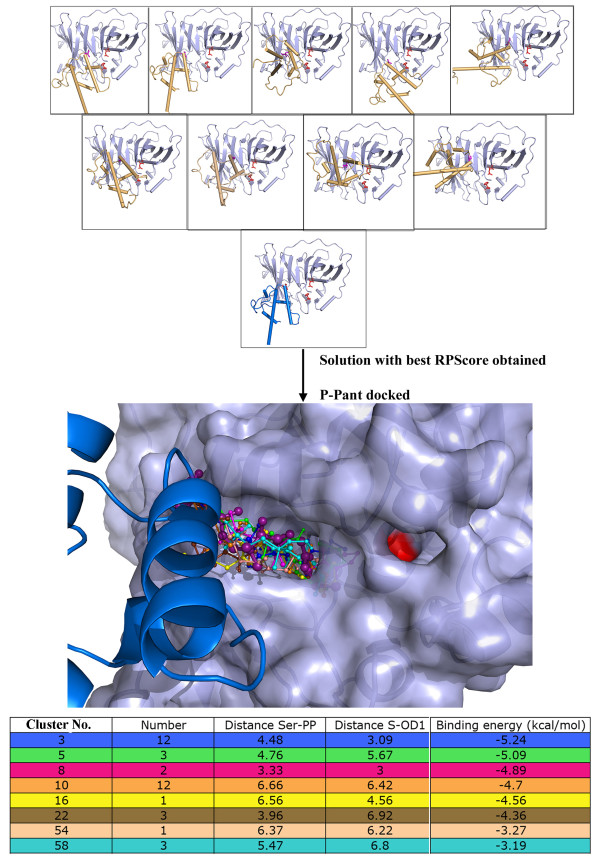
**The upper panel shows 10 solutions with positive RPScore obtained from protein-protein docking of apo-ACP onto DH domain.** Complex 2, which was selected for P-pant docking is shown with helices of ACP colored as blue. The ACP helices are colored orange for other solutions. The middle panel shows representative P-pant docking solutions from each cluster that satisfy distance constraints while bottom panel shows a table with these distances (Å) as well as corresponding binding energies (kcal/mol) for each of these clusters. Minimized final solution of holo-ACP:DH chosen for further substrate docking is shown in deep purple color with sticks in large thickness in middle panel.

The modeling of substrate bound holo-ACP:DH complex was carried out in several steps involving protein-protein docking of apo-ACP:DH complexes, docking of P-pant group, docking of substrate moiety and finally formation of required covalent bonds and energy minimization/refinement of the substrate bound complex as described in methods section (Figure 3 Additional file 1: Figures S1 and Figure S2). During docking of apo-ACP on DH domain, 10,000 complexes obtained from FTDOCK program [26] were re-ranked using residue-residue pair potentials, RPscore [27]. Out of these 10000 complexes, 355 complexes with a positive RPscore were selected for further analysis. These 355 complexes were further filtered using functional constraints. The functional requirement for catalysis by DH domain involved P-pant group attached to the Ser of ACP to reach the active site pocket of DH domain, thus bringing Ser in proximity with the residues which are located near the entrance to the active site cavity of the DH domain. Therefore, a distance cutoff of 5 Å between any atom of catalytic Ser of ACP and any atom of Phe 227 and Arg 275 of DH domain was used as functional constraint. In fact, site directed mutagenesis studies reported earlier have suggested involvement of these two residues from DH domain in recognition of ACP [19]. Interestingly, R275D mutation in erythromycin DH is known to significantly lower the yield of 6-dEB and based on these results, role of Arg 275 in ACP recognition has been proposed [19]. These functional constraints based filtering resulted in only 10 complexes satisfying the criteria. Detailed analysis of interface residues in these 10 apo-ACP:DH complexes indicated that, complex 2 (shown in Additional file 1: Figure S3) has not only high RPScore value, but also optimal orientation of Ser 46 for extension of the covalently attached P-pant group into the DH binding pocket. It also had most favorable interactions involving Asp 45 (ACP): Arg 275 (DH) and Leu 47 (ACP): Phe 227 (DH) pairs. Hence complex 2 was selected for the next step of P-pant docking to obtain holo-ACP:DH complex. The top panel of Figure 4 depicts the 10 solutions obtained from protein-protein docking of DH and apo-ACP domain. Complex 2 is represented with helices of ACP domain in blue color while in other solutions ACP domain is shown in orange color. The Ser of ACP is placed at the entrance of a deep cavity which leads to the catalytic His 44 and Asp 206 of DH domain [19].

After obtaining the biologically meaningful structural model for apo-ACP:DH complex, the next step was to model the holo-ACP:DH complex by docking of P-pant moiety as described in the methods section. The total of 250 bound conformations of P-pant obtained from AutoDock were clustered using a RMSD cut off of 2 Å and this resulted in 113 clusters with different binding energy values. The central panel in Additional file 1: Figure S4 shows the binding energy values and number of conformations for these 113 clusters obtained from P-pant docking. Since, functionally meaningful P-pant conformation should form covalent bond between Ser of ACP and its thiol group should be close to the catalytic residues of DH domain, the cluster corresponding to the lowest energy and highest population might not always be biologically meaningful. Therefore, distance between Oγ atom of Ser 46 (ACP) and phosphate of P-pant as well as distance between –SH group of P-pant and Oδ atom of Asp 206 (DH) was computed for representative conformations from each of these 113 clusters. The representative conformations from each of the clusters having the values of above-mentioned distances less than 7 Å but more than 3.5 Å have been depicted in middle panel of Figure 4, while the corresponding values of distances as well as binding energy are shown in the bottom panel. The cases where the distance was less than 3.5 Å between Ser of ACP and phosphate group of P-pant showed a steric clash between them (Clusters 3 and 8 shown in lower panel of Figure 4). After removal of such solutions, the cluster 5 was chosen in terms of minimum binding energy as well as distance constraint required for formation of covalent bond. As can be seen from the middle and lower panels of Figure 4, all the solutions satisfying the functional constraints fall into the same deep cavity leading to the catalytic residues and binding energy of these solutions varies in the range of −3.19 to −5.24 kcal/mol. The Additional file 1: Figure S4 shows these two distances for all clusters (upper panel) and the relative orientation of the conformation of P-pant group from cluster 5 with respect to Ser 46 (ACP) and Asp 206 (DH) (lower panel). Therefore, docked conformation corresponding to cluster 5 was chosen for modeling of holo-ACP:DH complex by formation of required covalent bond between Ser 46 and P-pant moiety. The middle panel of Figure 4 shows in stick representation the covalently attached P-pant conformation (larger thickness and deep purple color) obtained after minimization of the complete complex containing DH, ACP and P-pant moiety from cluster 5.

The next requirement was to dock the substrate moiety onto holo-ACP:DH complex such that the carboxyl group of the substrate will be at a position to form covalent bond with the S atom in P-pant and also the beta hydroxyl group of the substrate will be in close proximity of the catalytic residues (Asp 206 and His 44) in the DH domains. As described earlier in the methods section, the substrate for DH domain was docked as two separate fragments ( Additional file 1: Figure S1) and the required covalent bonds were made after selecting suitable solutions from these two AutoDock runs. The docking of the first fragment of the substrate ( Additional file 1: Figure S5) yielded 250 conformations belonging to 32 clusters (RMSD within cluster <2 Å) with different binding energy values. Additional file 1: Figure S5a shows the number of conformations, distance between Oδ atom of Asp 206 (DH) and beta hydroxyl of the substrate and also the distance between the carboxyl carbon of the substrate fragment and S atom of P-pant for each cluster. Inset to Additional file 1: Figure S5a shows the conformation of the substrate fragment 1 from the cluster 1 showing minimum values of these two distances also. Additional file 1: Figure S5b shows similar results from docking of the second fragment and distribution of distance between Cγ-Cδ in various docked clusters. The whole complex, hence obtained, was minimized after building the required bonds between ACP, P-pant and substrate fragments to obtain the final substrate bound holo-ACP:DH complex. Additional file 1: Figure S6a shows the final energy minimized structure for the substrate bound holo-ACP:DH complex. As can be seen, the substrate moiety as well as a portion of the P-pant group enters into a deep cavity which harbors the catalytic residues of the DH domain. In order to further validate the results from the current study, the bound conformation of the substrate obtained from the current study was compared with the bound conformation of the mechanistic inhibitor 3-decynoyl-N-acetylcysteamine which has been crystallized in complex with FabA (PDB ID 1MKA), the type II FAS DH enzyme from E. coli [9]. Additional file 1: Figure S6b shows the superposition of 1MKA on the DH domain in substrate bound holo-ACP:DH complex obtained from the current study. As can be seen, in both cases the ligands bind into the narrow tunnel of DH leading to the catalytic residues ( Additional file 1: Figure S6b) and the bound conformation of the mechanism-based inhibitor 3-decynoyl-N-acetylcysteamine is also very similar to the conformation of the bound substrate in the ACP:DH complex. Additional file 1: Figure S6b shows the complete docked substrate backbone in green while the backbone atoms of the mechanism-based inhibitor have been depicted in blue color. The only difference in the conformation lies at the α-β positions because the inhibitor covalently binds to the catalytic His residue with cis conformation of the α-β bond[9], while the substrate for EryDH4 binds non-covalently in trans conformation of the corresponding α-β bond ( Additional file 1: Figure S6b). It shows that the binding site obtained by docking results is same as that observed by earlier experimental studies.

Detailed analysis of the substrate bound holo-ACP:DH complex indicated that the charged residues in DH domains interact with hydroxyl moieties of the substrate while the hydrophobic residues were in contact with methyl groups. The P-pant moiety was exposed to solvent to a large extent and therefore, fewer charged or polar residues had interaction with the P-pant moiety in the apo-ACP:DH complex. Table 1 shows the list of residues in DH domain, which interact with the ACP, P-pant and the substrate moiety. We also wanted to investigate whether the residues of DH domain which interact with ACP, P-pant and substrate moieties remain conserved in other modular PKS clusters. Therefore, the evolutionary conservation of these residues was checked. Additional file 1: Figure S7 shows the multiple sequence alignment (MSA) of DH domains from different modular PKS cluster with the interacting residues marked using arrow symbol. As can be seen from the MSA in Additional file 1: Figure S7, 10 out of 13 residues (His44, Pro53, Gly54, Ser55, Leu161, Tyr168, Asp206, Ala209, Pro226, Val274) interacting with substrate, 9 out of 13 residues (His44, Leu51, Val52, Gly54, Pro81, Leu82, Leu225, Pro226, Phe227) interacting with phosphopantetheine and both residues (Phe227 and Arg275) interacting with ACP show good conservation. The conservation of these residues indicates that the interactions of DH domain with ACP, P-pant and substrate obtained in holo-ACP:DH complex modeled for eryPKS can also be used to predict the interacting residues in case of DH domains from other clusters whose structures are not available yet.

**Table 1:** Contacts of DH domain with bound substrate and ACP in holo-ACP:DH complex

**Number**	**Contacts with substrate**	**Contacts with Phosphopantetheine**	**Contacts with ACP**
1	HIS 44	HIS 44	PHE 227
2	PRO 53	LEU 51	ARG 275
3	GLY 54	VAL 52	
4	SER 55	GLY 54	
5	LEU 161	LEU 78	
6	GLN 164	GLN 79	
7	TYR 166	ARG 80	
8	TYR 168	PRO 81	
9	ASP 206	LEU 82	
10	ALA 209	TYR 166	
11	GLN 210	LEU 225	
12	PRO 226	PRO 226	
13	VAL 274	PHE 227	

**Figure 5:**
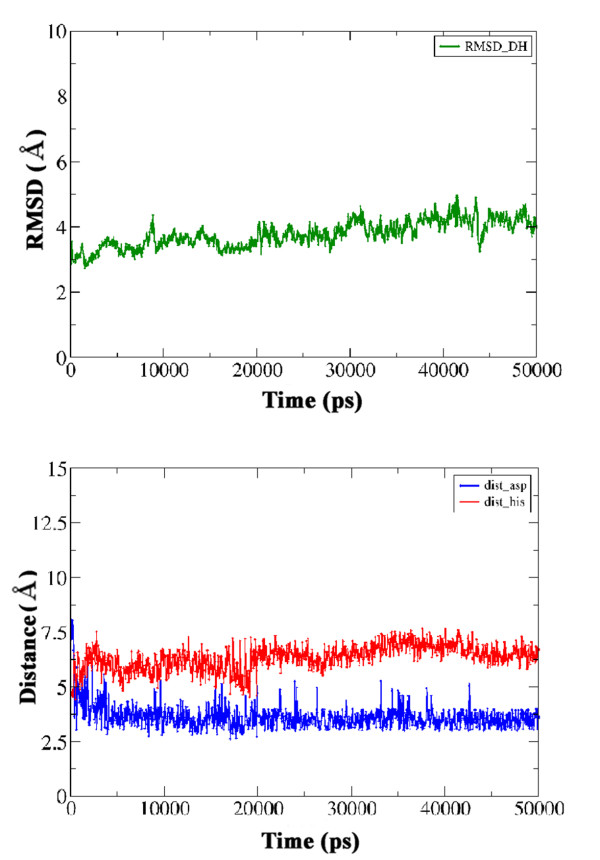
(**a) RMSD (Å) vs. Time (ps) plots for the substrate bound holo-ACP DH complex over 50 ns trajectory. (b)** Distance (Å) between Oδ atom of Asp 206 (DH) and the beta-hydroxyl group of the substrate (blue), alpha carbon of the substrate and Nϵ of His (red) over 50 ns MD trajectory.

**Figure 6:**
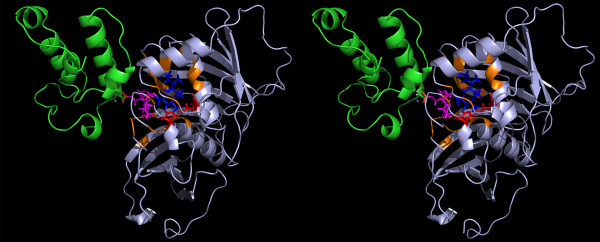
**The figure depicts the final solution obtained after 50 ns MD simulations in stereo view.** The DH (lightblue) and ACP (green) domains are depicted as cartoon representation while the sticks represent final conformation of P-pant (magenta) and substrate (blue). The catalytic His and Asp are depicted in red sticks.

Since, the protein-protein as well as protein-ligand docking used to model this substrate bound holo-ACP:DH complex did not incorporate flexibility of protein residues, MD simulations were carried out in the explicit solvent environment for a period of 50 ns to further refine the substrate bound holo-ACP:DH complex. This helped in incorporating protein as well as ligand flexibility and also to check the stability of the solutions obtained by docking studies. Detailed structural analysis of the conformations of the ACP:DH complexes sampled during the 50 ns indicated that the entire complex showed a backbone RMSD in the range of 3.0 to 4 Å with respect to the starting structure used for MD simulations (Figure 5a). This suggested that there were no large scale changes in the relative orientation of ACP domain with respect to the DH domain and the structure obtained from docking was stable. Analysis of the P-pant attached substrate conformations over the entire 50 ns trajectory also indicated that Oδ atom of Asp 206 (DH) remained within 4.0-5.0 Å of the beta-hydroxyl group of the substrate, while the distance between alpha carbon of the substrate and Nϵ of His was within 6.0-7.0 Å (Figure 5b). This suggests that bond between Cα and Cβ which is dehydrated to a trans double bond during catalysis by DH indeed remains in close proximity of the catalytic residues of the DH domain throughout the 50 ns simulation. Figure 6 shows the stereo view of the final complex obtained after 50 ns MD simulation and the contacting residues with P-pant and substrate moiety are depicted in orange color. Additional file 1: Figure S8 shows the comparison of the substrate binding tunnel in the crystal structure of the substrate free DH domain ( Additional file 1: Figure S8a) with the tunnel in the substrate bound DH domain ( Additional file 1: Figure S8b) after removal of the P-pant bound substrate moiety. As can be seen, there are subtle changes in the structure of the DH domain which facilitates substrate binding by opening of the tunnel. In fact, similar substrate binding tunnel is also seen in other DH domains. Akey *et al*[18] have suggested that substrate binding to curacin DH would also involve such opening of tunnel as was seen in the current docking and MD simulation studies on erythromycin DH domain.

**Figure 7:**
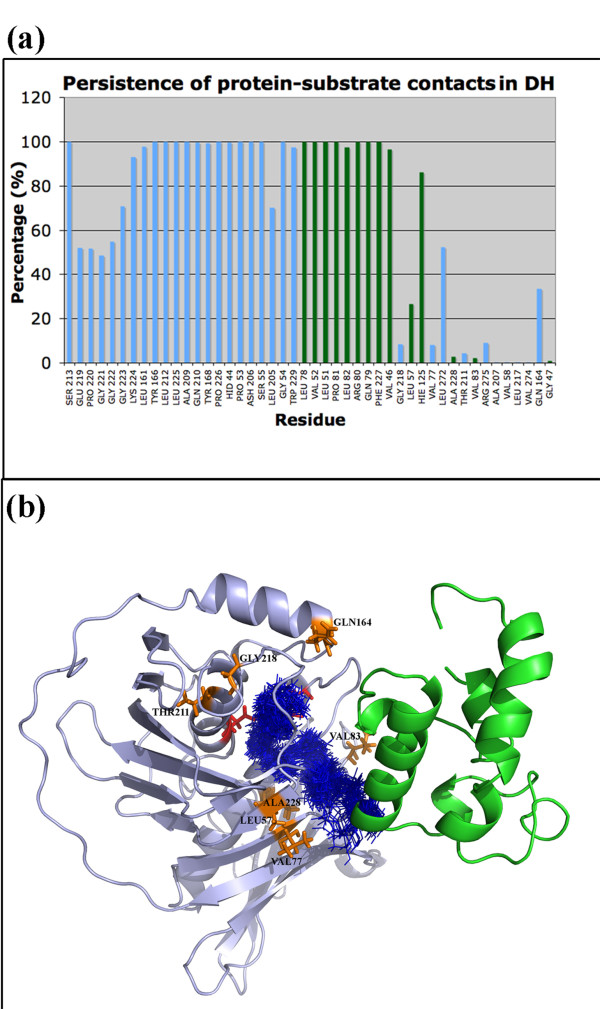
**(a) The figure shows the percentage (y-axis) of time each residue shown on x-axis was involved in contact with DH domain.** The residues forming contacts with P-pant have been shown as green bars while those in contact with substrate have been shown as blue bars.**(b)** The figure depicts (blue colored line representation) conformations of P-pant and substrate extracted every 1 ns from the MD trajectory of duration 50 ns for DH domain. The residues which show contacts with the P-pant or substrate moiety for less than 40% of the simulation time have been shown as orange sticks.

Detailed analysis of the 50 ns MD trajectory indicated that the P-pant attached substrate moiety indeed sampled multiple conformations within the active site tunnel of the DH domain. Therefore, it was interesting to investigate whether the binding pocket residues identified by docking persisted throughout the 50 ns simulations and if new contacts were formed during the MD simulation. Figure 7a shows the percentage of time for which the various residues of DH domain remained in contact with the P-pant as well as substrate group during the 50 ns simulation. Thus, different residues interact with P-pant and the substrate moieties for different durations. It is interesting to note that, only 26 residues showed interaction with P-pant or substrate for more than 80% of the simulation time. On the other hand, out of 18 residues of DH domain showing contact for less than 60% of the time, 14 were involved in contacts with the substrate while only 4 residues showed contacts with P-pant moiety. The residues showing higher persistence times are likely to be more critical for substrate and P-pant binding. These results have interesting implications for identifying substrate binding residues of DH domains by experimental studies. Figure 7b shows the snapshots of conformations taken up by P-pant and substrate moiety extracted from the 50 ns trajectory at an interval of 1 ns. The residues interacting with the P-pant-substrate moiety for less than 40% but more than 10% of the time have also been depicted. These residues might be interacting with only a small population of the conformations of the P-pant and substrate moiety. Thus incorporation of flexibility by MD simulations has given a dynamic picture of the interactions between the substrate and DH domain.

### Modeling and analysis of substrate bound holo-ACP:KR complex

The modeling of substrate bound holo-ACP:KR complex was carried out using a similar protocol (Figure 3) as that used for ACP:DH complex involving modeling of apo-ACP:KR complex by protein-protein docking, generation of holo-ACP:KR complex by docking of P-pant group and finally docking of the substrate group. However, the substrate moiety was docked in a single step because of its smaller size unlike the case of docking of substrate on DH domain.

**Figure 8:**
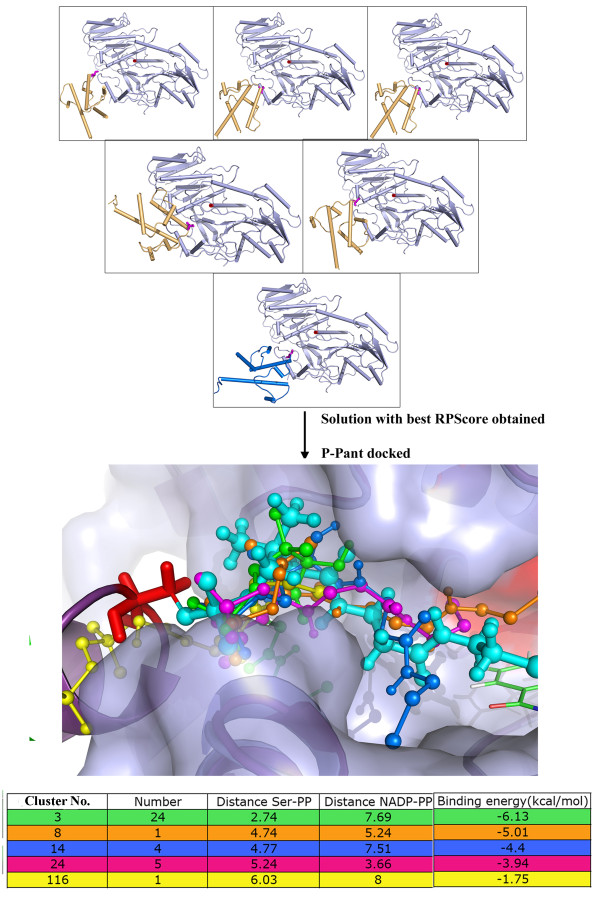
**The upper panel of the figure shows 6 solutions with positive RPScore obtained from protein-protein docking apo-ACP onto KR domain.** Complex 1, which was selected for P-pant docking is shown with helices of ACP colored as blue. The ACP helices are colored orange for other solutions.The middle panel shows representative P-pant docking solutions from each cluster that satisfy distance constraints while bottom panel shows a table with these distances (Å) as well as corresponding binding energies (kcal/mol) for each of these clusters.Minimized final solution of holo-ACP:KR chosen for further substrate docking is shown in deep purple color with sticks in large thickness in middle panel.

Docking of apo-ACP and KR domains by FTDOCK yielded 10000 complexes which were re-ranked using residue-residue pair potentials (RPscore). The set of 267 complexes obtained with a positive RPscore were further analyzed to identify the complexes satisfying functional constraints. The functional requirement for catalysis by KR domain necessitates the 20 Å long P-pant group attached to the Ser of ACP to reach the active site pocket of KR domain. Therefore, the complexes were filtered with a distance constraint of 20 Å between catalytic Ser 46 of ACP and the catalytic Tyr 1813 of KR domain. It may be noted that, in case of apo-ACP:DH docking study, distance constraints between Ser 46 of ACP and residues on DH domain were used because experimental studies had suggested involvement of corresponding residues in ACP recognition. However, no such information was available about the interacting residues involved in binding of ACP to the KR domain. Therefore, only the 20 Å distance constraint between Cα atom of Ser 46 of ACP and Cα atom of catalytic Tyr 1813 of KR was used to filter the complexes having positive RPscore. This functional constraint yielded six complexes that satisfied the criteria and the best scoring complex was chosen as the final apo-ACP:KR complex. The top panel in Figure 8 shows the six complexes satisfying functional constraints obtained from apo-ACP:KR docking, while Additional file 1: Figure S9a shows the final apo-ACP:KR complex selected for P-pant docking to obtain holo-ACP:KR complex. The analysis of interface residues of the apo-ACP:KR complex obtained from this docking study revealed favorable interactions involving Arg 1857 (KR): Asp 45 (ACP) and Phe 1856 (KR): Leu 47 (ACP) pairs ( Additional file 1: Figure S9b). It is interesting to note that interacting residue pairs in ACP:KR interface are also identical to the interacting pairs in ACP:DH complex, as discussed earlier. Similar interaction interface involving Asp:Arg pair is also seen in complexes of ACP with ACP synthetase (AcpS) [7,28]. This suggests that the functional constraint based docking study has successfully identified interaction interface for apo-ACP:KR complex. Analysis of apo-ACP:KR complex also revealed that Ser 46 of ACP was placed at the entrance of the shallow solvent exposed cavity on the surface of the KR domain while the opposite end of this cavity harbored the NADPH binding site adjacent to the catalytic residues.

In order to model the holo-ACP:KR complex, the P-pant moiety was docked on the final apo-ACP:KR complex obtained from protein-protein docking. Docking of P-pant moiety by AutoDock on the apo-ACP:KR complex resulted in 250 bound conformations belonging to 150 different clusters (RMSD cutoff of 2 Å). The lower left panel in Additional file 1: Figure S10 shows the number of conformations as well as binding energy values for each of these 150 clusters, while the top panel shows the distance between phosphate of P-pant and Ser 46 of ACP as well as the distance of the thiol group of P-pant from the KR bound NADPH for each of these clusters. Since, functionally meaningful P-pant conformation should form covalent bond between Ser 46 of ACP and its thiol group should be close to the catalytic residues of KR domain, the cluster corresponding to the lowest energy and highest population need not be biologically meaningful. Therefore, distance between Oγ atom of Ser 46 of ACP and phosphate of P-pant as well as distance between –SH group of P-pant and NADPH was used as functional constraints for filtering functionally meaningful bound conformation for P-pant group. The solutions with the values of above mentioned distances less than 8 Å but more than 3.5 Å have been depicted in middle panel of Figure 8 while the corresponding values of distances as well as binding energy are shown in the bottom panel. As can be seen from Figure 8**,** all these solutions fall into the same shallow cavity leading to the catalytic residues and binding energy of these solutions varies in the range of −1.75 to −6.13 kcal/mol. Even though cluster 8 was best solution in terms of minimum binding energy as well as distances, it had a steric clash between P-pant group and catalytic Tyrosine. Thus, the next best solution cluster 14 was chosen for modeling the holo-ACP:KR complex by formation of required covalent bond between Ser 46 and P-pant moiety. The middle panel of Figure 8 shows the stick representation (larger thickness and cyan color) of the P-pant conformation after minimization of the complete complex containing KR, ACP and P-pant moiety from cluster 14. Although, in the docked conformation of the P-pant moiety, the distance between NADPH and thiol group of P-pant was around 7 Å ( Additional file 1: Figure S10), after minimization the distance was reduced to 3.6 Å. In the holo-ACP:KR complex, the P-pant group was located in the shallow cavity which also contained NADPH binding site.

**Figure 9:**
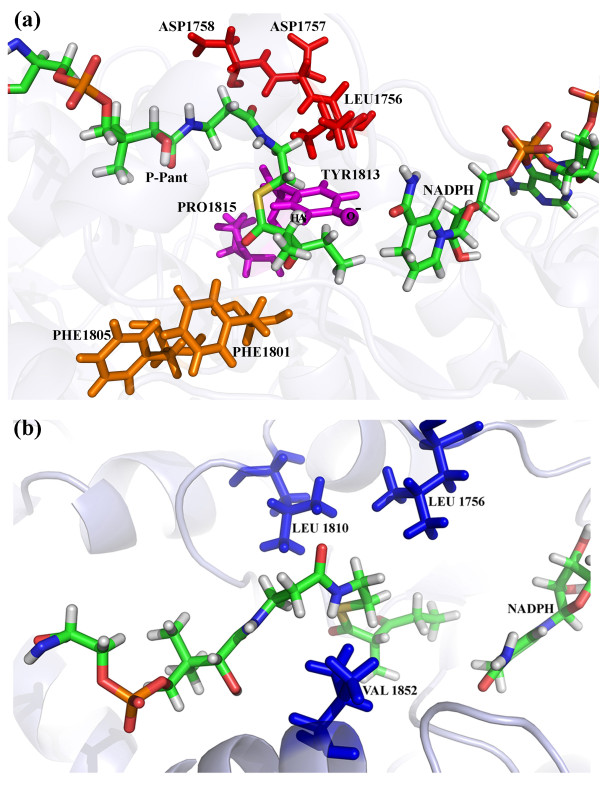
**The figure depicts the residues involved in determination of stero-chemistry of reduction as well as the epimerization at alpha position. (a)** The residues of LDD motif are depicted in red sticks, catalytic Tyr 1813 and Pro1815 are shown in magenta color while the Phe1805 and Phe1801 are shown in orange representation. The hydrogen atom of substrate and O^-^ of Tyr1813 which are hypothesized to be involved in epimerization have been depicted as spheres. **(b)** The residues Val1852, Leu1810 and Leu1756 which form hydrophobic pocket around substrate are depicted in blue sticks.

In order to build the substrate bound holo-ACP:KR complex, the diketide substrate moiety was docked onto the holo-ACP:KR complex subject to the constraint that, the terminal carboxyl carbon of the substrate will be covalently bonded to the S atom in P-pant and also the beta keto group of the substrate will be in close proximity of the catalytic residues in the KR domain. The various bound conformations of the substrate obtained from AutoDock were grouped into 15 clusters (RMSD within a cluster <2 Å) with different binding energy values. Additional file 1: Figure S11 shows the number of conformations as well as distances corresponding to the functional constraints in each of the 15 clusters. The inset in Additional file 1: Figure S11 shows the docked conformation of the substrate from cluster 12 which had minimum values for the two distance constraints. The lowest energy docked conformation from cluster 12 was selected for modeling the substrate bound holo-ACP:KR complex by formation of required covalent bond between P-pant and substrate. The whole complex, hence obtained, was energy minimized and the final energy minimized substrate bound holo-ACP:KR complex is shown in Additional file 1: Figure S12. As can be seen, the substrate moiety and the P-pant group binds into ashallow cavity exposed to the surface and span the region from entrance of the cavity till the NADPH binding site, which is located at the other end of this cavity. Figure 9 shows the conformation of the P-pant bound substrate and its orientation with respect to the key stereo specificity determining residues in the catalytic pocket of the KR domain. In addition to the catalytic residues, it has also been proposed that LDD motif interacts with substrate and is responsible for determining stereo-specificity in case of B-type KRs [20]. In addition, the second aspartate of the LDD motif as well as the Phe1801 residue is proposed to determine stereo specificity of keto-reduction by experimental studies. In contrast, the conserved Tryptophan is responsible for guiding the substrate into the binding cavity in case of A-type KRs. In case of B-type KRs the conserved Tryptophan is replaced by a Phenylalanine residue (F1805). It has been proposed that, F1805 blocks the substrate binding in orientations similar to A-type KRs and hence the substrate interacts with LDD motif and enters from other side of the cavity. As can be seen from Figure 9a, in the substrate conformation obtained from our docking studies, the Leu of this LDD motif (Leucine 1756 depicted in red) is in close proximity of the hydrophobic region of the P-pant moiety and is away from F1805. Keatinge-Clay & Stroud have also proposed that Tyr 1813 is liberated out of the helix αF by the helix-breaking residue Pro 1815. This imparts higher mobility to Tyr 1813 which allows it to abstract hydrogen from alpha position on the polyketide to bring about epimerization [20] at alpha-position. After epimerization Val 1852 from lid helix as well as Leu 1810 can interact with epimerized methyl group via hydrophobic interactions and hence, stabilize it after the KR domain brings about the epimerization (Figure 9b) [20]. As can be seen from Figure 9b, Tyr 1813 and Val 1852 are located on either side of the substrate. Thus, the substrate binding site identified by the current docking and MD simulations is also consistent with the mechanistic details of the proposed mechanism of epimerization by KR domain at alpha carbon of the substrate.

Analysis of the apo-ACP:KR complex indicated that the charged residues in KR domains formed contacts with hydroxyl or carboxyl moiety of the substrate while the hydrophobic residues were observed to interact with methyl groups. In case of P-pant moiety, the charged residues in KR domain interact with the charged phosphate group as well as carboxyl atoms while the non-polar residues interact with the methyl groups of P-pant. Table 2 shows the corresponding interacting residues and they have been marked on the multiple sequence alignment (MSA) of KR domains to observe their evolutionary conservation ( Additional file 1: Figure S13). As can be seen, 5 out of 8 residues (Ser1800, Leu1810, Tyr1813, Trp1839, Gly1840) interacting with substrate moiety are evolutionarily conserved ( Additional file 1: Figure S13). On the other hand, only 2 out of the 11 residues (Leu1810, Tyr1813) interacting with the phosphopantetheine group show good conservation, while none of the residues interacting with ACP was conserved. In fact the binding site on the KR domain is located on the surface and many of these interacting residues in KR domain belong to loop regions on the structure. It is possible that the residues on KR domain would have co-evolved with corresponding interacting residues on the cognate ACP domains. The recent studies on the KR domains from hedamycin and actinorhodin clusters from Type II PKSs also showed that the residues interacting with substrate as well as ACP differed within these two domains [14,15].

**Table 2:** Contacts of KR domain with bound substrate and ACP in holo-ACP:KR complex

**Number**	**Contacts with substrate**	**Contacts with Phosphopantetheine**	**Contacts with ACP**
1	SER 1800	ALA 1807	ARG 1855
2	PHE 1801	PRO 1808	PHE 1856
3	LEU 1810	LEU 1810	ARG 1858
4	TYR 1813	TYR 1813	HIS 1859
5	TRP 1839	THR 1841	GLN 1901
6	GLY 1840	MET 1847	
7	THR 1841	VAL 1852	
8	PHE 1856	ARG 1855	
9		PHE 1856	
10		ARG 1858	
11		HIS 1859	

**Figure 10:**
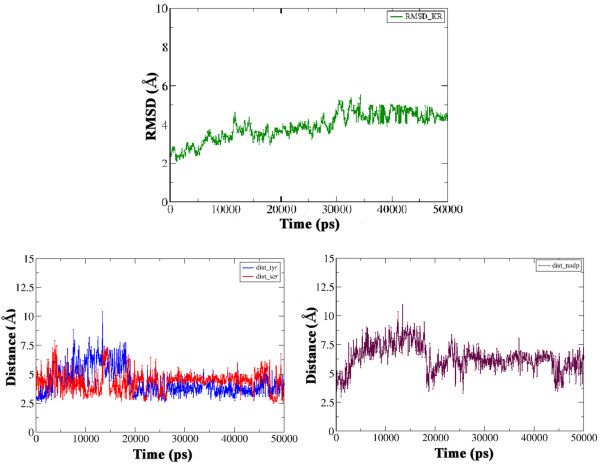
**RMSD (Å) vs. Time (ps) plots for the substrate bound holo-ACP KR complex over 20 ns MD trajectory.** Distance (Å) between between keto group at beta position of the substrate and hydroxyl of Tyr 1813 as well as the side chain oxygen of Ser 1800 over 20 ns MD trajectory. The lower right panel shows the variation of the distance (Å) between NADPH and beta carbon of the substrate over the MD trajectory.

**Figure 11:**
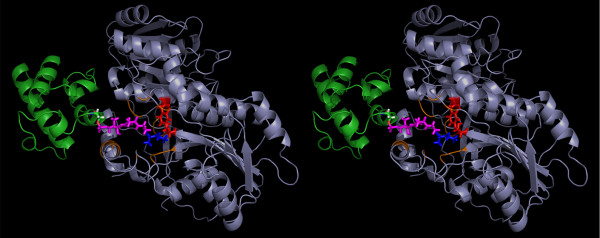
**The figure depicts the final solution obtained after 50 ns MD simulations in stereo view.** The KR (lightblue) and ACP (green) domains are depicted as cartoon representation while the sticks represent final conformation of P-pant (magenta) and substrate (blue). The catalytic Tyr and Ser are depicted in red sticks.

**Figure 12:**
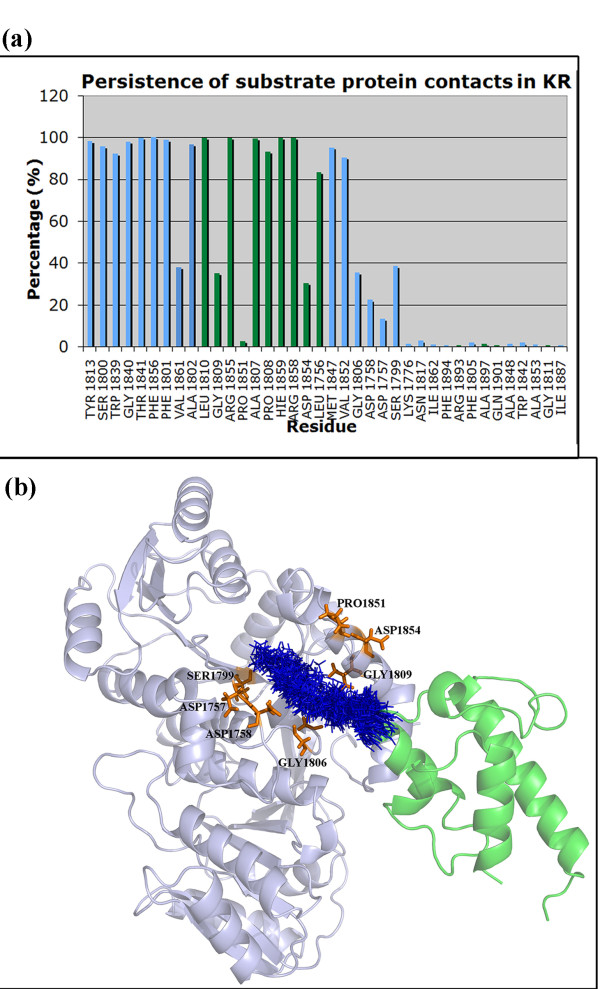
**(a) The figure shows the percentage (y-axis) of time each residue shown on x-axis was involved in contact with KR domain.** The residues forming contacts with P-pant have been shown as green bars while those in contact with substrate have been shown as blue bars.**(b)** The figure depicts (blue colored line representation) conformations of P-pant and substrate extracted every 1 ns from the MD trajectory of duration 50 ns for KR domain. The residues which show contacts with the P-pant or substrate moiety for less than 40% of the simulation time have been shown as orange sticks.

The substrate bound holo-ACP:KR complex obtained from docking and energy minimization studies was further refined using molecular dynamics simulations in the explicit solvent environment for a period of 50 ns. Detailed analysis of the conformations obtained from 50 ns simulations indicated that the entire complex showed a backbone RMSD in the range of 3.0 to 5.0 Å from the starting structure. The conformation of the bound substrate and its orientation with respect to the catalytic residues of KR domain was also analyzed to investigate whether the binding pose of the substrate identified by docking and energy minimization was stable during the explicit solvent MD simulation. As can be seen from the lower left panel in Figure 10, the distance between keto group at beta position of the substrate and hydroxyl of Tyr 1813 as well as the side chain oxygen of Ser 1800 remains within 3.0 to 6.0 Å throughout the 50 ns trajectory. It has been proposed that during catalysis by KR domain, Lys 1776 is involved in activation of Tyr 1813 to a general acid so that it can donate its proton to the carbonyl oxygen at beta position after hydride transfer from NADPH [5]. Analysis of the orientations of Lys 1776 over the 50 ns trajectory indicated that it remained in proximity of Tyr 1813 throughout the simulation. The lower right panel in Figure 10 shows the variation of the distance between NADPH and beta carbon of the substrate over the MD trajectory. As can be seen this distance increases from 3.0 Å in the starting structure to as high as 7.5 Å within 5 ns, but towards the last half of the simulation it comes to a value of 5.0-6.0 Å. These results suggest that, the substrate binding site identified by the current docking and MD simulation studies is consistent with the proposed mechanism for catalysis by KR domain. Figure 11 shows stereo view of the final complex obtained after 50 ns MD simulation and the contacting residues are depicted in orange color. Figure 12a shows the persistence of contacts between the KR domain and the P-pant as well as substrate moiety during the 50 ns simulation. As can be seen, only 17 contacting residues showed interaction with P-pant or substrate for more than 80% of the simulation time. Out of the 21 residues showing contact for less than 60% of the time, 14 were involved in contacts with the substrate while only 7 showed contact with P-pant moiety. Figure 12b shows the snapshots of conformations taken up by P-pant and substrate moiety extracted from the 50 ns trajectory at an interval of 1 ns.

**Figure 13:**
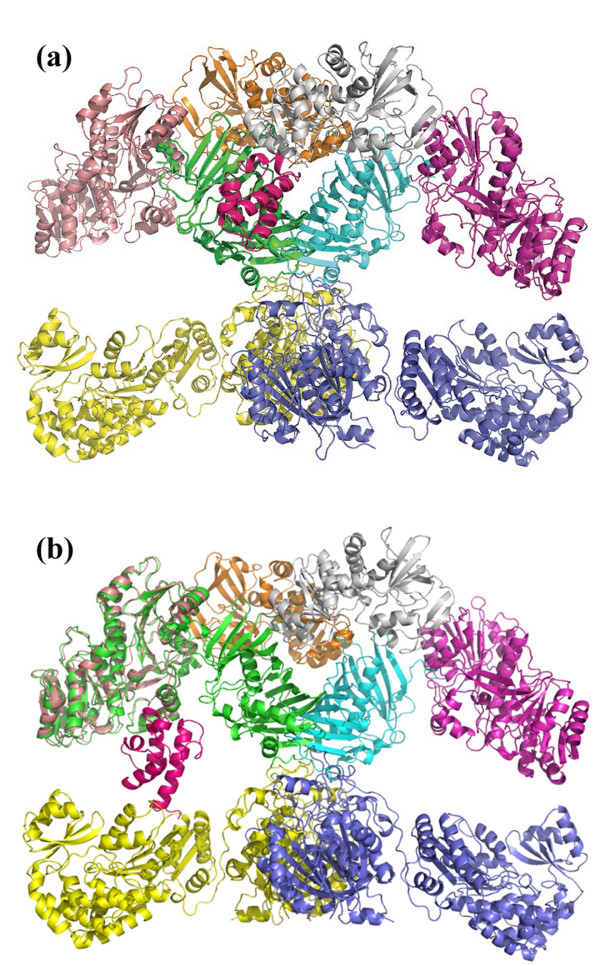
**(a) The figure shows the DH-ACP complex transformed on to the structural model for module 4 of erythromycin PKS.** There are no steric clashes of ACP with any other domain in the module. **(b)** The figure shows the KR-ACP complex transformed on to the structural model for module 4 of erythromycin PKS. There are no steric clashes of ACP with any other domain in the module.

The holo-ACP:DH as well as holo-ACP:KR complexes obtained by our study gave the position of ACP domain with respect to DH and KR domains but in a modular PKS the ACP would bind to a complete module structure comprising other domains also. Thus, it was necessary to check if the predicted site of ACP binding on each of these domains is also available in the three dimensional structure of the complete PKS module or occluded by other catalytic domains. Since, no complete module structure is available for PKS, the structure for complete module 4 of erythromycin PKS was modeled using FAS as template using SBSPKS web-server [29]. The apo-ACP:KR and apo-ACP:DH complexes obtained from protein-protein docking studies were transformed on to this structural model for complete module 4 of erythromycin PKS. As can be seen from Figure 13a and 13b**,** even in the complete module structure, ACP can bind to the same sites as predicted by docking and there are no steric clashes of ACP with any other domain in the module. It is interesting to note that the ACP binding site on KR domain is located in a void surrounded by KR, DH, AT and KS domains.

## Conclusion

In this study, the binding of DH and KR domains to their cognate apo-ACP domains has been modeled using protein-protein docking methods. The energetically favorable solutions obtained from docking studies have been further filtered to satisfy functional constraints like proximity of catalytic Ser of ACP to active sites of DH and KR domains. The apo-ACP:DH and apo-ACP:KR complexes generated by this approach have been utilized to model holo-ACP:DH and holo-ACP:KR complexes by docking of P-pant moiety and substrate fragments. The protein-ligand docking for modeling holo-ACP complexes has also been carried out based on functional constraints of covalent linkage between Ser of ACP, P-pant and substrate moiety. Since, the protein-protein and protein-ligand docking do not incorporate protein flexibility, the holo-ACP complexes obtained from docking studies have been subjected to unrestrained molecular dynamics simulations in explicit solvent environment for a period of 20 ns. Interestingly, the holo-ACP:DH and holo-ACP:KR complexes were found to be stable even during unrestrained MD simulations.

This study gives an insight into how ACP binds to DH and KR domains and also how the substrate moieties are swung into the active sites by P-pant arm of ACP. It was found that substrate enters into a deep tunnel in case of DH domain while in case of KR domain the substrate binds in a shallow cavity exposed to the surface. Based on these modeling studies, the specific residues on DH and KR domains involved in interaction with ACP, P-pant and substrate moiety have been identified. The substrate binding site on DH domain identified from the current modeling study has been validated by comparison with the crystallographically determined bound conformation of the mechanism based inhibitor of FabA protein. Similarly, in case of KR domain, the substrate is positioned in proximity to the known sequence motifs responsible for epimerization as well as stereo-chemical control, as explained earlier. Even though, the current modeling study has been carried out for DH and KR domains from erythromycin synthase, analysis of conservation profile of binding pocket residues indicate that the results might be extrapolated to DH and KR domains of other PKS clusters also.

## Methods

The DH and KR domains carry out their catalytic activity on a polyketide intermediate which is covalently attached to the P-pant moiety of the holo-ACP[1]. Therefore, the protocol for *in silico* modeling of substrate bound apo-ACP:DH and apo- ACP:KR complexes involved several steps which are illustrated in the flowchart depicted in Figure 3. First, apo-ACP:DH and apo-ACP:KR complexes were generated by protein-protein docking approach using FTDOCK [26]. Subsequently, the respective acylated P-pant moieties, the substrates for the DH and KR domains respectively, were docked to the apo-ACP:DH and apo-ACP:KR complexes by protein-ligand docking approach using AutoDock [30]. However, the acylated P-pant groups which are cognate substrates for the DH and KR domains used in this study are large chemical moieties with too many freely rotatable bonds. Therefore, instead of docking them as a single ligand moiety, protein-ligand docking was carried out in several steps by docking the P-pant group and fragments of substrates separately as shown in Additional file 1: Figure S1. The various solutions obtained from protein-ligand docking were filtered using functional constraints like proximity of docked ligand to the catalytic residues of DH and KR domains, covalent bonding distance between phosphate of P-pant and Ser of ACP, covalent bonding distance between thiol group of P-pant and substrate fragments etc. Details of various steps involved in modeling substrate bound holo-ACP:DH and holo-ACP:KR complexes are described below.

### Docking of homology models of cognate ACPs onto crystal structures of DH and KR domains

The cognate ACP for the crystal structure of DH domain of erythromycin synthase is the ACP from module 4 while ACP from module 1 of erythromycin is the cognate ACP for the crystal structure of the KR domain. Since, no crystal or NMR structures were available for these two ACPs, NMR structure (PDB ID 2JU2) of the ACP from module 2 of erythromycin[31] PKS was used as template for modeling the structures of these cognate ACPs for DH and KR domains. Homology modeling of these ACPs was carried out using Modeller version 9v8 [32].

Docking of the structural models of apo ACP domains without P-pant onto the crystal structures of DH and KR domain was carried out using the protein-protein docking software FTDOCK version 2 [26]. During the protein-protein docking, the larger DH or KR domain was treated as receptor and kept static, while the smaller ACP domain was kept mobile. The DH domain was projected onto 176 × 176 × 176 grid with a grid step of 0.696 Å and a global surface thickness of 1.3 Å. The grid size for KR domain was 192 × 192 × 192 with a grid step of 0.703 Å. Both for DH-ACP and KR-ACP docking studies, the rotation angle step was 12°, a total of 9,240 rotations were evaluated in total and three best complexes were selected from each rotational step. FTDOCK runs were carried out with electrostatic potential turned on. Finally, a total of 10000 top scoring complexes were given by FTDOCK as possible solutions. These 10000 complexes were further re-ranked using RPScore [27] module of FTDOCK package which uses a scoring function similar to residue based statistical pair potentials. Few hundred high scoring complexes based on positive RPScore values were further filtered based on functional or biological constraints like proximity of catalytic sites of DH or KR domains to the P-pant attachment site on the ACP domain.

### Modeling of holo-ACP:DH and holo-ACP:KR complexes

In order to model complexes of holo-ACP with DH and KR domains, it was necessary to dock the phosphopantetheine (P-pant) group on the final structures of apo-ACP:DH and apo-ACP:KR complexes obtained by functional constraints based filtering of the high scoring solutions given by FTDOCK. However, in these complexes, ACP blocks the entrance to the active site pockets of DH and KR domains. Therefore, during the docking of phosphopantetheine group, ACP was removed from these complexes and docking was carried out on DH and KR domains alone. The docking of phosphopantetheine group onto the DH and KR complexes was carried out by using the program AutoDock version 4 [30]. The coordinates for the phosphopantetheine (P-pant) moiety were obtained from the NMR structure of the holo-ACP from Spinach (PDB ID 2FVA) [33]. The structure 2FVA contained a stearic acid bound phosphopantetheine attached to the catalytic Ser of ACP. The hydrogens were added to the ligand and Gasteiger charges [34] were assigned to various atoms of the ligand. During docking, all bonds other than peptide bonds were kept rotatable. The docking grid consisting of 126 × 126 × 126 points with a grid spacing of 0.375 Å was centered on the catalytic site of DH (His 44) or KR (Tyr 1813) domain and covered a large portion of DH or KR complex. Docking was carried out using the Lamarckian genetic algorithm (LGA) as conformational search method [30]. The docking parameters were set to 27,000 generations, 25,00,000 energy evaluations, and 250 docking runs and default values were used for the other parameters. The final set of 250 receptor bound conformations for P-pant obtained from docking studies on DH and KR complexes were clustered using a cluster radius of 2 Å RMSD.

The various different P-pant bound DH and KR domains obtained from the AutoDock analysis were transformed onto the apo-ACP:DH and apo-ACP:KR complexes. This resulted in ACP:DH and ACP:KR complexes in which P-pant group was bound in different conformations and orientations. In the holo-ACP:DH and holo-ACP:KR complexes, the phosphate of the P-pant group should be covalently bonded to Ser of ACP and the terminal -SH group of P-pant should be proximal to the catalytic residues of DH or KR domains. Out of the 250 solutions obtained by docking, P-pant conformations which had the minimum distance between the S atom of P-pant and Cα of respective catalytic residues in DH/KR domains as well as between phosphate of P-pant and Ser of ACP were selected using an in-house Perl script. The P-pant bound ACP:DH and ACP:KR complexes which satisfied the above mentioned functional constraints were used to model holo-ACP:DH and holo-ACP:KR complexes by forming the required covalent bonds between the Ser of ACP and the phosphate of P-pant. The holo-ACP:DH and holo-ACP:KR complexes obtained by this approach were further energy minimized using CVFF forcefield and Insight II package.

### Modeling of substrate bound holo-ACP:DH and holo-ACP:KR complexes

In order to model holo-ACP:DH and holo-ACP:KR complexes bound to their native substrates, the cognate substrate moieties for the DH and KR domains were docked onto the holo-ACP:DH and holo-ACP:KR complexes obtained by P-pant docking. Complexes were energy minimized after formation of covalent bonds, as mentioned earlier. The substrate for KR domain was a diketide while the substrate for DH domain was (2R,3R,4R,6R,7S,8S,9R)-3,7,9-trihydroxy-5-oxo-2,4,6,8 tetramethylundecanoate [19]. The structural models for these substrate moieties were built using Biopolymer module of Insight II. The longer substrate for DH domain which consisted of 10 carbon atoms was modeled starting from the coordinates of the 18 carbon stearic acid from 2FVA and making relevant substitutions of hydrogen atoms by hydroxyl or methyl groups as per desired stereochemistry. The structures of cognate substrates generated by Insight II were also energy minimized in isolation prior to their docking on the holo-ACP:DH and holo-ACP:KR complexes.

In case of the substrates for DH domain, the number of freely rotatable bonds was larger. Therefore, the 10 carbon atom substrate was docked as two separate fragments one after another. Additional file 1: Figure S1 shows the fragments of DH substrate which were docked in two different steps. As in the case of P-pant docking, the ACP domain was removed from the ACP:DH or ACP:KR complex for facilitating access of the substrate to the catalytic pocket of DH or KR domain. However, the P-pant group was bound to the DH and KR domain and it was kept rigid during the docking of the substrate moieties for DH and KR domains. The docking was carried on using AutoDock 4 and all other parameters were same as in the case of P-pant docking. The various bound conformations of substrates or substrate fragments obtained from AutoDock 4 were analyzed in terms of their proximity to the catalytic residues of DH or KR domains and distance between the terminal carboxyl of the substrate to the –SH group of the P-pant with which the substrates should form covalent bonds. The docked conformations of the substrates which satisfied these functional constraints were selected for further analysis. After selecting the final conformation of the substrates, the P-pant and substrate fragment bound DH or KR domains were transformed onto the apo-ACP:DH or apo-ACP:KR complexes by superposition of the DH or KR domain only. This was done in order to get coordinates of ACP domain with respect to substrate and P-pant bound DH or KR domain. The required bonds between –SH group of P-pant and carboxyl group of substrate as well as bonds between two different substrate fragments of DH domains were created. Thus, the complete structure of the substrate bound holo-ACP:DH or holo-ACP:KR domain obtained by this approach was energy minimized first by CVFF forcefield of Insight II and subsequently by AMBER package [35].

### MD simulations on substrate bound holo-ACP:DH and holo-ACP:KR complexes

The structural models of substrate bound apo-ACP:DH and apo-ACP:KR complexes obtained from protein-protein docking and protein-ligand docking contained an unusual side chain residue where catalytic Ser of ACP was covalently bonded to acylated P-pant moiety. Antechamber module of AMBER 9 [36] was used to assign charges and other molecular mechanics forcefield parameters to this chemically modified Ser moiety ( Additional file 1: Figure S2a and Figure S2b). Coordinate and topology files for these substrate bound holo-ACP:DH and holo-ACP:KR complexes were generated using xleap module of AMBER 9. The force field used was ff03 [37] for amino acids and TIP3P water model was used to solvate the protein. The water box extended 14 Å beyond the coordinates of the outer most atom of the protein-protein complexes along all three axes. The solvated structures were minimized using steepest descent minimization with a convergence criterion of 0.001 kcal/mole/Å as RMS gradient of potential energy. After minimization, MD simulations were carried out on the solvated protein-protein complexes using a time step of 1 fs and SHAKE [38] was used for constraining any bonds involving heavy atoms and hydrogen. The temperature of the system was raised to 300 K using NVT ensemble over a period of 200 ps in order to distribute the kinetic energy added into the system due to heating to 300 K among all degrees of freedom. Temperature coupling was performed using Langevin dynamics with a collision frequency 3 ps^-1^. The reference pressure was set to 1 atm using NPT ensemble over a further period of 200 ps. The equilibration was carried on till the density (~1 g/cm^3^) as well as temperature (~300 K) became stable. The pressure was scaled using isotropic position scaling with a pressure relaxation time of 1 ps. The production simulation was carried out for 50 ns for both the substrate bound holo-ACP:DH as well as holo-ACP:KR complexes using NPT ensemble. The non-bonded cutoff of 8 Å was used for van der Waals interactions. Particle Mesh Ewald (PME) summation [39] was used to compute long-range electrostatic interactions. The cutoff of 8 Å is used to define the range within which the direct sum computation of electrostatics will occur and beyond the cutoff reciprocal sum calculation of electrostatics is performed.

During the simulation, the coordinates were saved at an interval of 1 ps. Various analyses on the MD trajectories were performed using ptraj module of AMBER 9 as well as other in-house Perl scripts.

### Calculation of the persistence of contacts during MD simulations

A set of 1000 structures were extracted from 50 ns trajectory with conformations taken at an interval of 50 ps. The residues contacting the substrate and P-pant moiety were calculated for each of these structures. The contacting residues included those where any atom of the residue lied within a cutoff distance of 6 Å from any atom of P-pant and substrate moieties. For each of the contacting residues, the percentage of structures in which the corresponding contact was present was reported.

## Authors’ contributions

DM and SA designed research. SA performed the computations and analyzed data. DM and SA analyzed data and wrote the manuscript. All the authors read and approved the final manuscript.

## Supplementary Material

Additional file 1 Supplementary figures. Click here for file
