# Supplementary material for: Modeling holo-ACP:DH and holo-ACP:KR complexes of modular polyketide synthases: a docking and molecular dynamics study
Source: BMC Struct Biol. 2012 May 28;12:10. doi: 10.1186/1472-6807-12-10 (PMC3422181; doi:10.1186/1472-6807-12-10)
Supplement: Additional file 1 — Supplementary figures. [file 1472-6807-12-10-S1.pdf]

SUPPLEMENTARY FIGURES

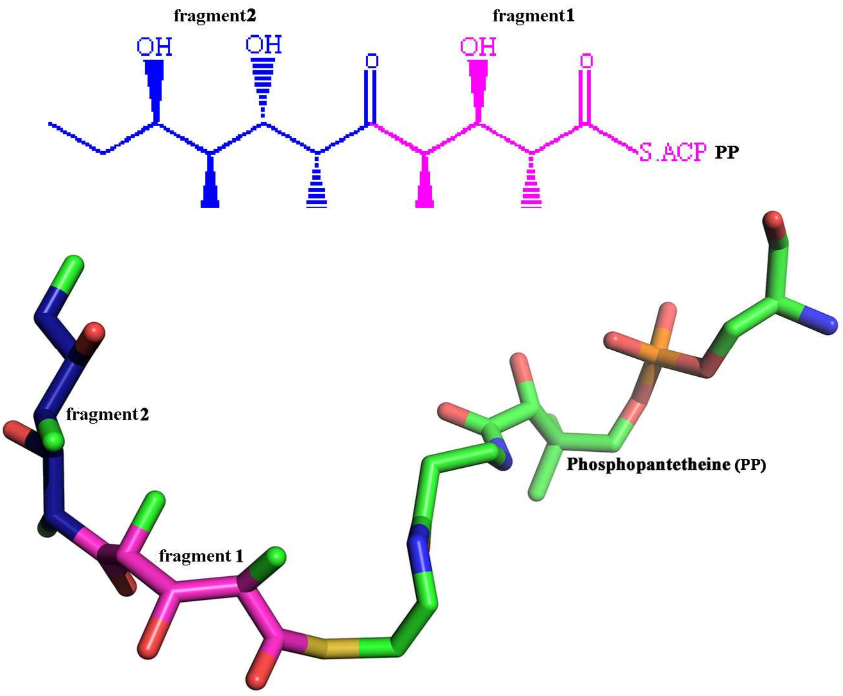

Figure S1

The substrate for DH domain (2R,3R,4R,6R,7S,8S,9R)-3,7,9-trihydroxy-5-oxo-2,4,6,8 tetramethylundecanoate) was docked as two separate fragments shown in magenta and blue respectively. The fragment 1 is connected to phosphopantetheine by a thioester linkage.

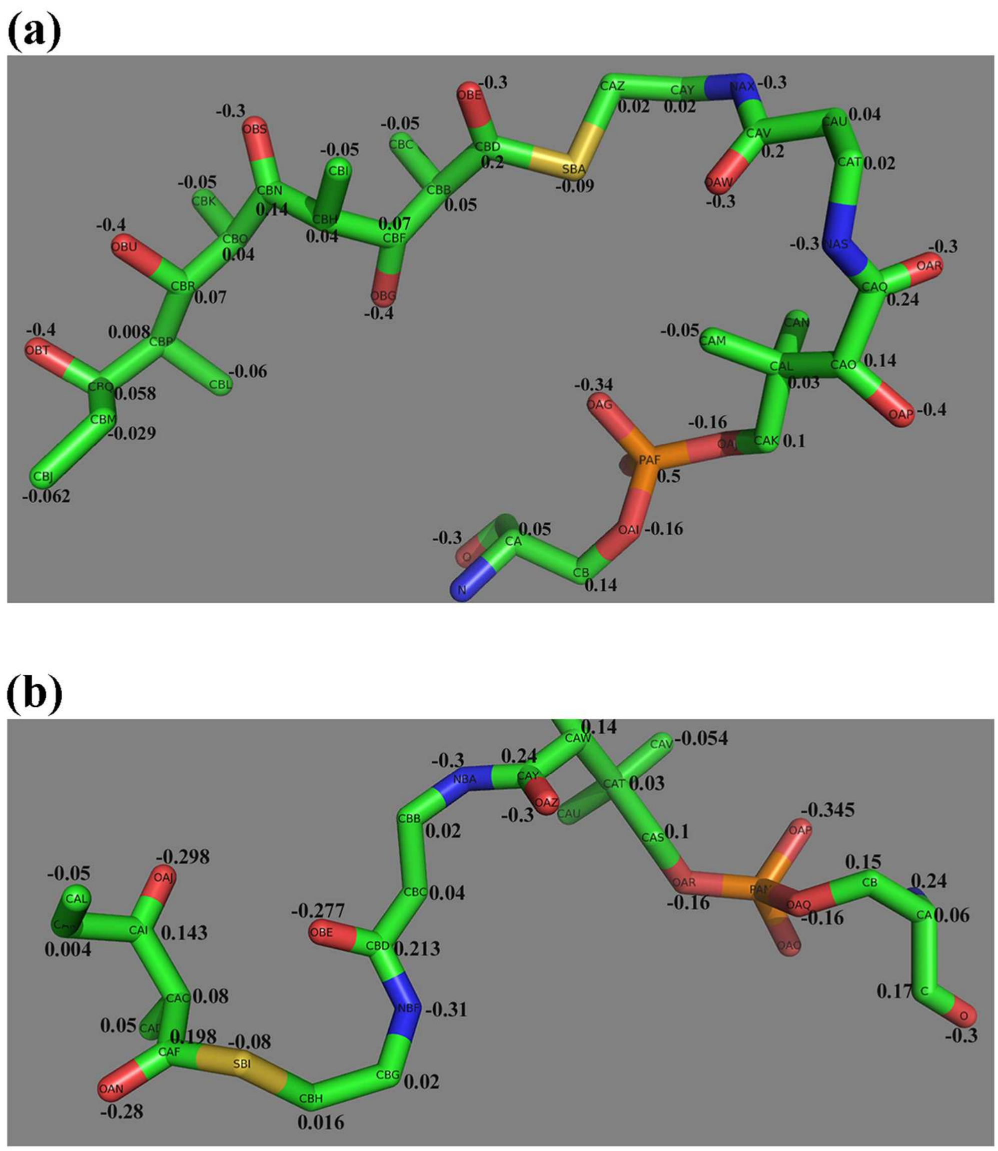

Figure S2

(a) Charges assigned to different atoms by antechamber module of AMBER9 in Ser-P-Pant-substrate moiety, which was bound to DH domain. Substrate was (2R,3R,4R,6R,7S,8S,9R)-3,7,9-trihydroxy-5-oxo-2,4,6,8 tetramethylundecanoate.

(b) Charges assigned to different atoms in Ser-P-Pant-substrate ligand for KR domain by antechamber module of AMBER9.

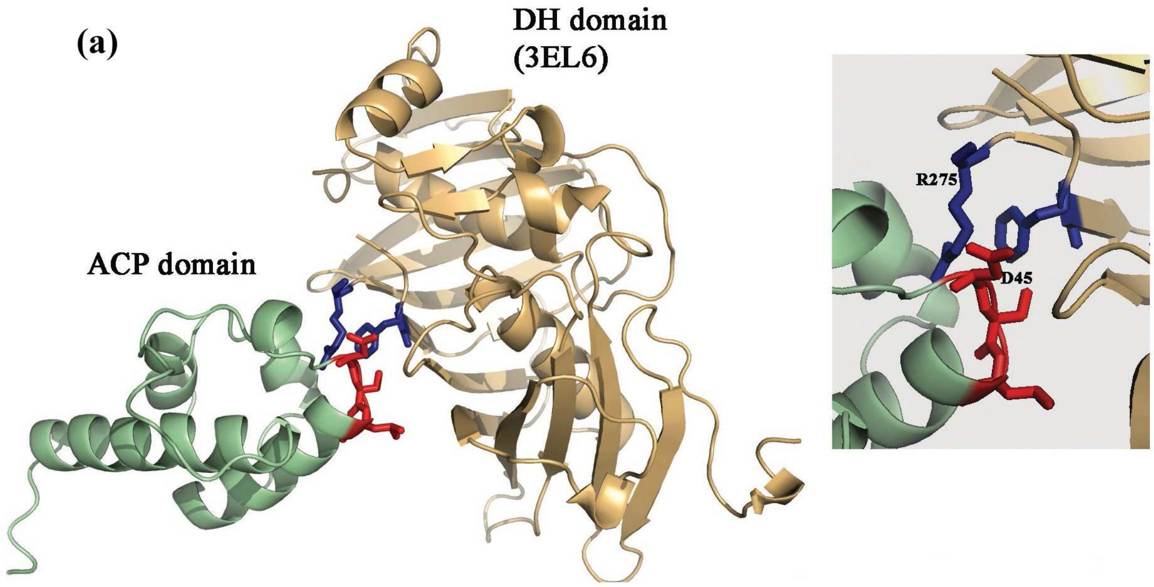

Figure S3

The favorable interaction of R275 (DH) and D45 (ACP) is shown in the Complex 2 of apo DH-ACP obtained by FTDOCK

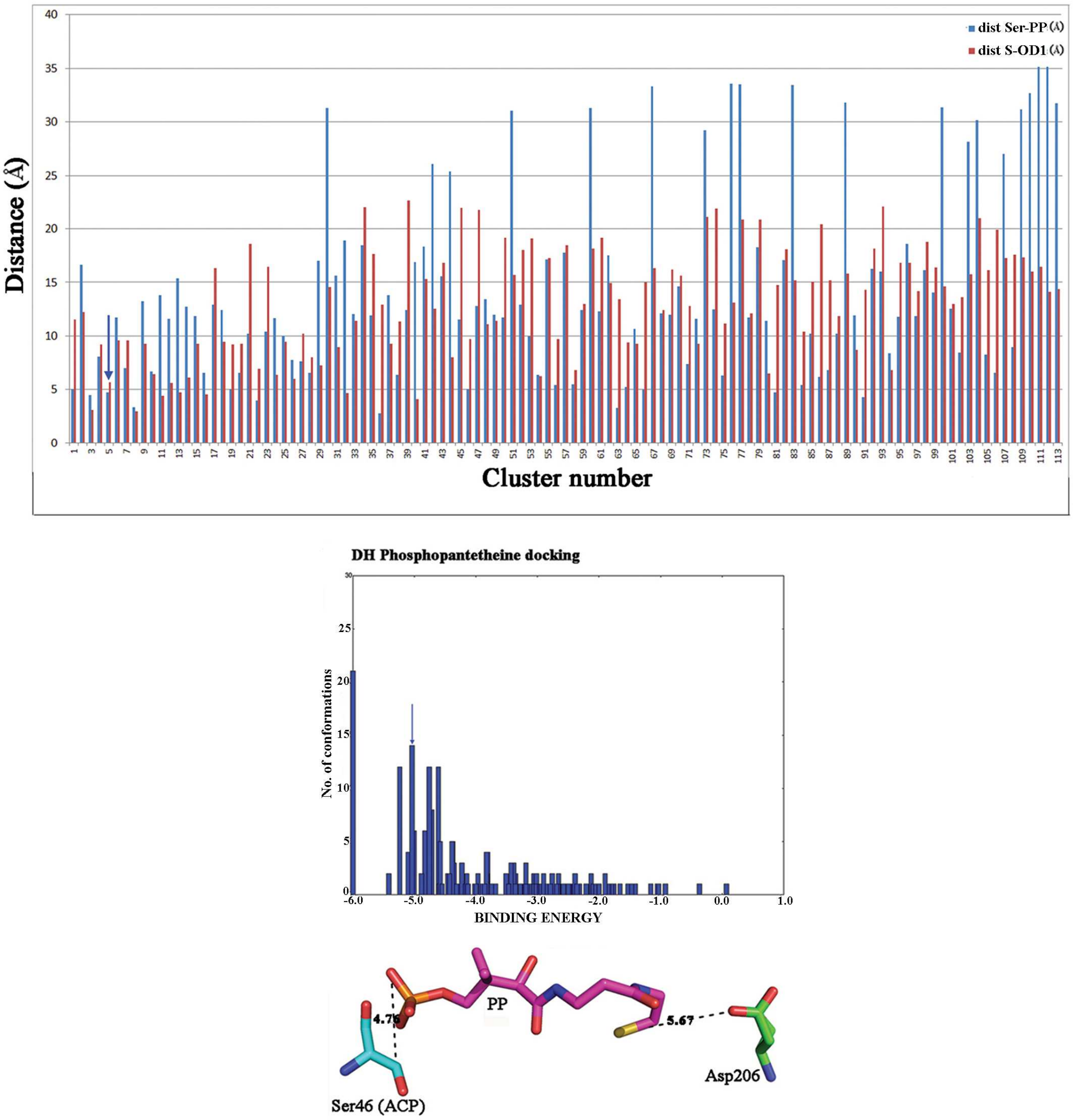

Figure S4

Upper panel shows the distance between gamma-OH of Ser 46 (ACP) and gamma-OH of phosphate in P-pant (PP) (blue) as well as distance between –SH group of P-pant and Oδ atom of Asp 206 of DH (red) for representative conformations from each of these 113 clusters. The central panel shows the binding energy values and number of conformations for these 113 clusters obtained from P-pant docking. Lower panel shows the relative orientation of the P-pant (PP) group from cluster 5 with respect to Ser 46 (ACP) and Asp 206 (DH).

(a)

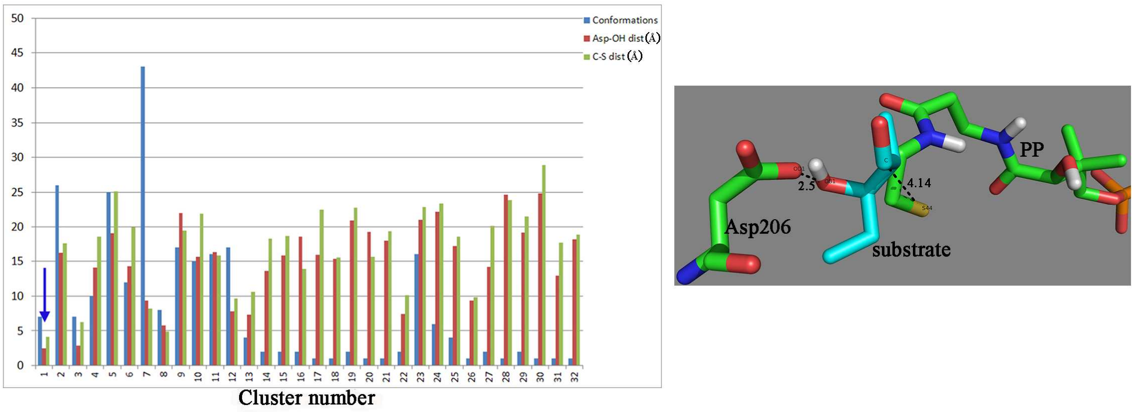

(b)

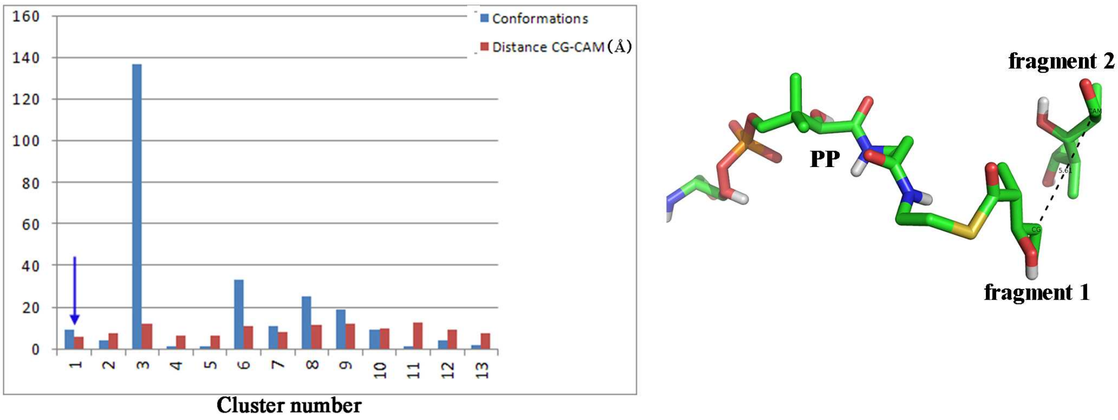

Figure S5

(a) The figure shows the number of conformations (blue), distance between Oδ atom of Asp 206 (DH) and beta hydroxyl of the substrate (red) and also the distance between the carboxyl carbon of the substrate fragment and S atom of P-pant (PP) for each cluster (green). Inset to figure shows the conformation of the substrate fragment 1 from the cluster 1 showing minimum values of these two distances also.

(b) Results from docking of the second fragment and distribution of distance between Cγ-Cδ in various docked clusters. Inset to figure shows the conformation of the substrate fragment 2 from the cluster 1 showing minimum values of the distance also.

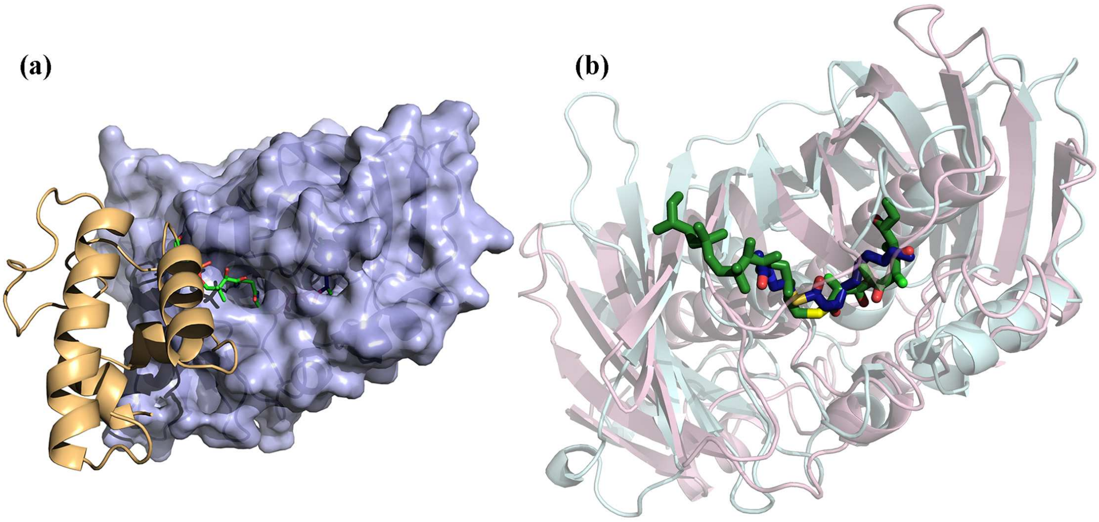

Figure S6

a) The final energy minimized structure for the substrate bound holo ACP-DH complex. The P-Pant and substrate have been depicted using stick models.

b) Superposition of 1MKA with mechanism based inhibitor bound (blue) onto the DH domain in substrate bound holo ACP-DH complex (substrate shown in green) obtained from the current docking study. The yellow colored atoms of stick models in both cases depict sulphur while the red color atoms show oxygen.

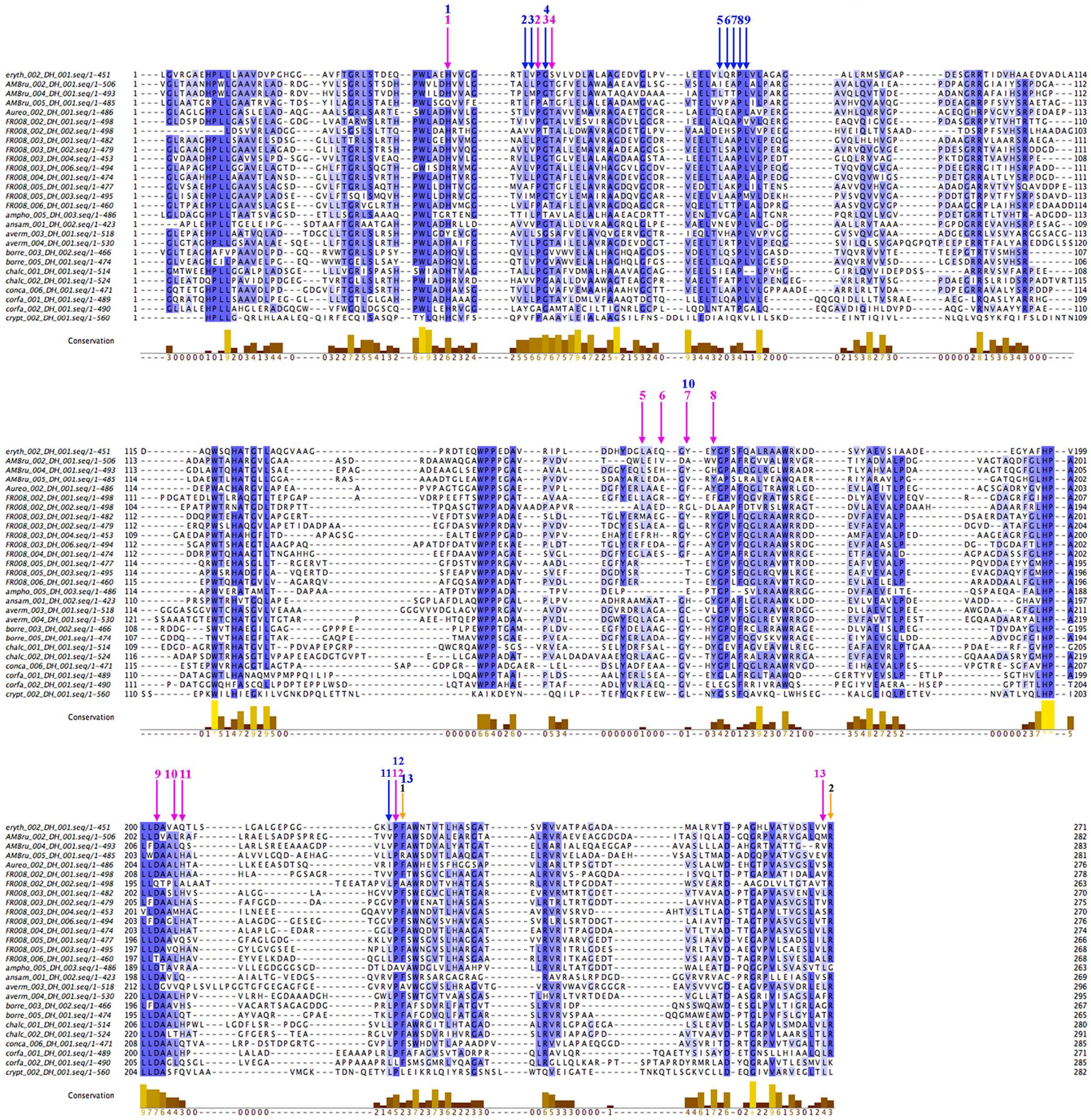

Figure S7

The figure shows the multiple sequence alignment for DH domains and the residues interacting with ACP (yellow), Phosphopantetheine (blue) and substrate (magenta) in the substrate bound holo-ACP-DH complex have been marked. The numbers above the arrows indicate the row numbers in Table 1 which lists the identity and the number of the corresponding residues.

(a) (b)

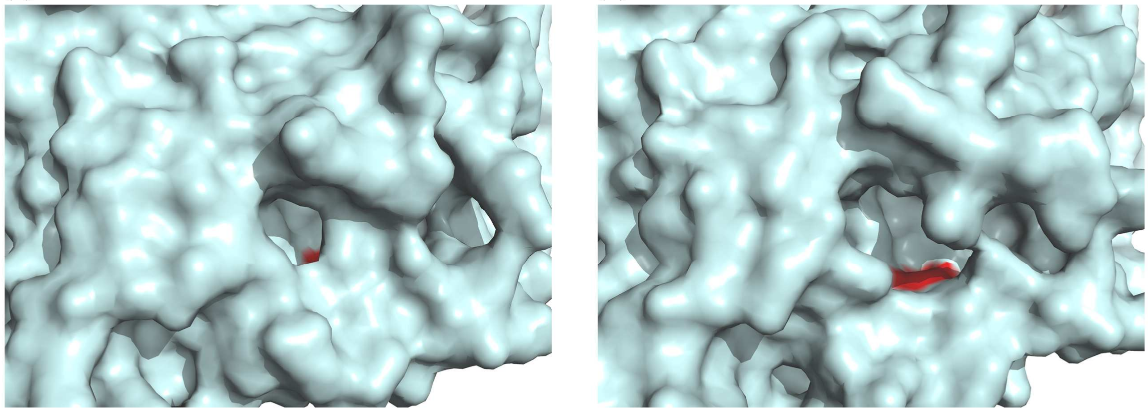

Figure S8

Comparison of the substrate binding tunnel in the (**a**) crystal structure of the substrate free DH domain with the (b) tunnel in the substrate bound DH domain complex obtained at the end of 20ns MD simulation after removal of the P-pant bound substrate moiety.

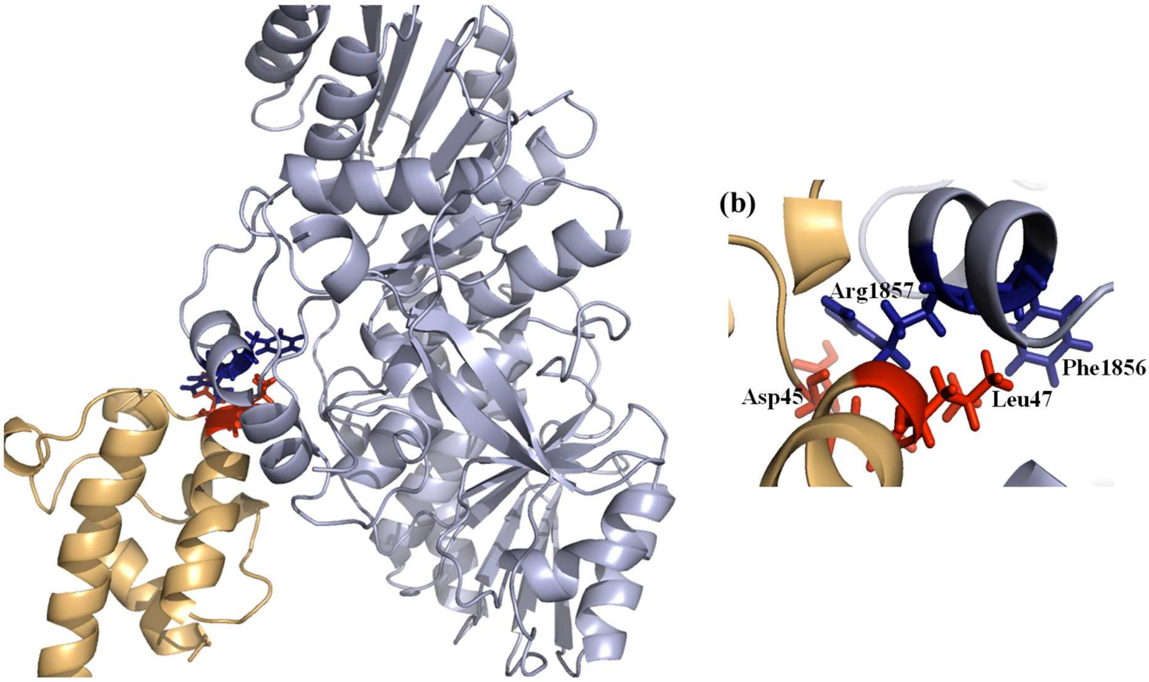

Figure S9

(a) The apo-ACP-KR complex obtained by FTDOCK

(b) The favorable interaction of R1857 (DH) and D45 (ACP) is shown in the Complex 1 of apo KR-ACP obtained by FTDOCK

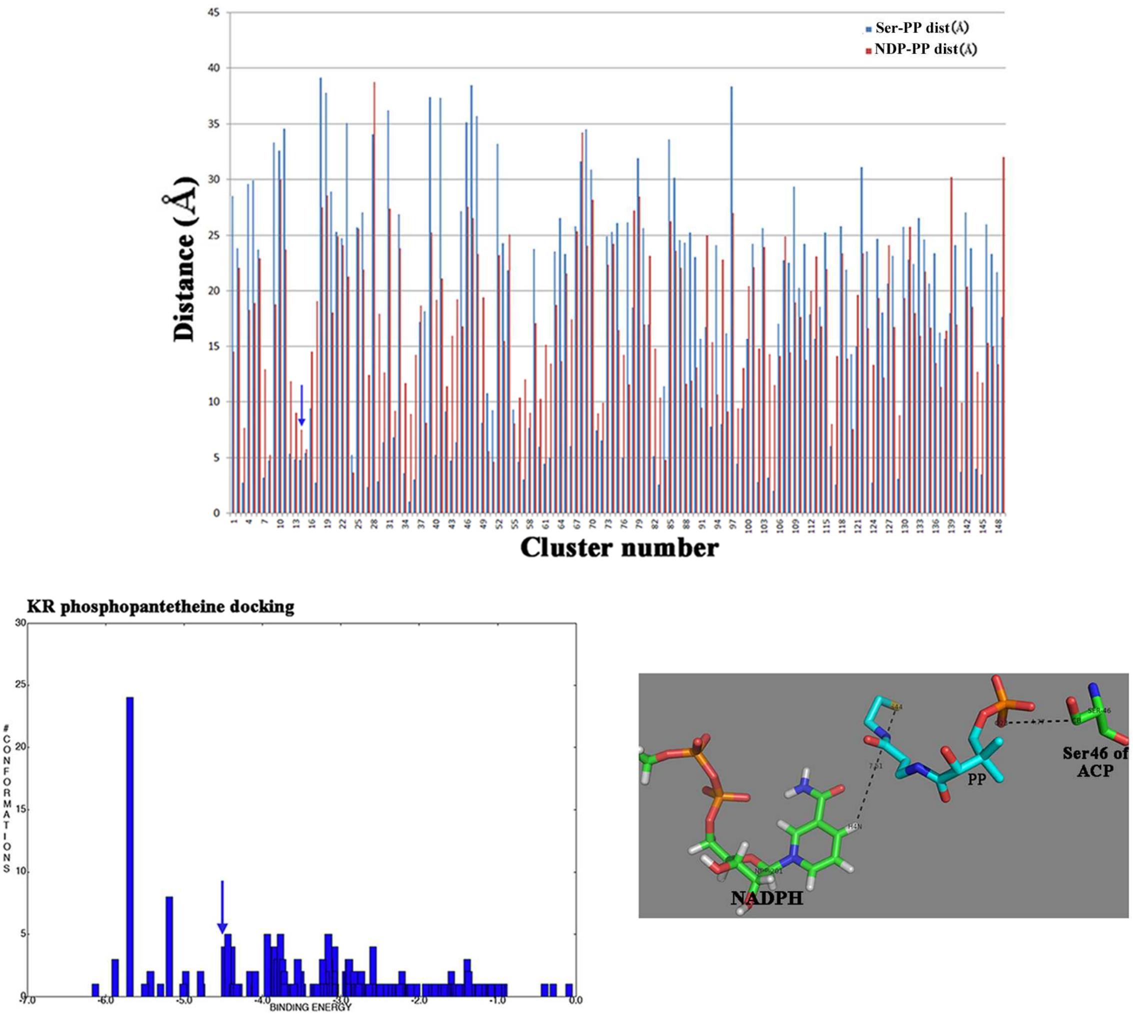

Figure S10

The lower left panel shows the number of conformations as well as binding energy values for each of these 150 clusters, while the top panel shows the distance between phosphate of P-pant (PP) and Ser 46 of ACP (blue) as well as the distance of the thiol group of P-pant from the KR bound NADPH (red) for each of these clusters. Lower right panel shows the relative orientation of the P-pant (PP) group from cluster 14 with respect to Ser 46 (ACP) and NADPH (KR).

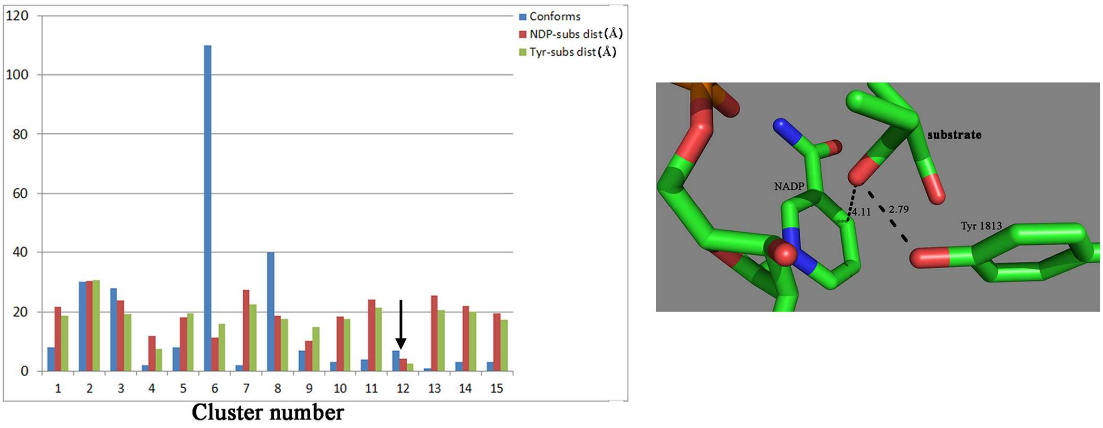

Figure S11

The left panel shows the number of conformations as well as distances corresponding to the functional constraints in each of the 15 clusters obtained on substrate docking. The inset shows the docked conformation of the substrate from cluster 12 with had minimum values for the two distance constraints.

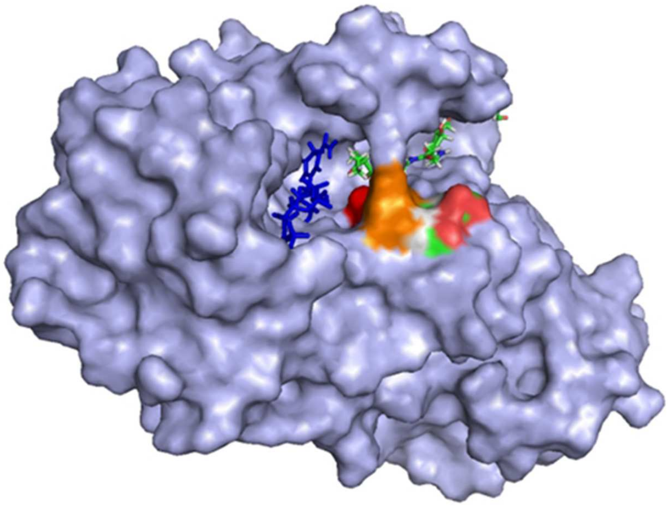

Figure S12

Holo-ACP-KR complex post MD simulation. The figure depicts the proximity of substrate to LDD motif (Leucine 1756 depicted in orange). Catalytic Tyr 1813 (red) can interact with it and help in epimerization at alpha position.

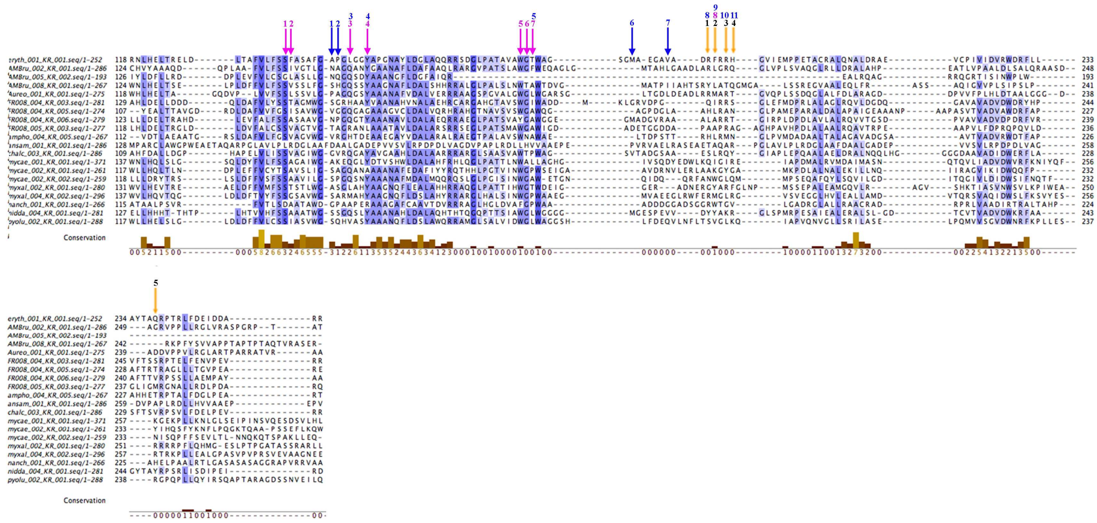

Figure S13

The figure shows the multiple sequence alignment for KR domains and the residues interacting with ACP (yellow), Phosphopantetheine (blue) and substrate (magenta) in the substrate bound holo-ACP-KR complex have been marked. The numbers above the arrows indicate the row numbers in Table 2 which lists the identity and the number of the corresponding residues.
